# Natural Variation in the Distribution and Abundance of Transposable Elements Across the *Caenorhabditis elegans* Species

**DOI:** 10.1093/molbev/msx155

**Published:** 2017-05-09

**Authors:** K.M. Laricchia, S. Zdraljevic, D.E. Cook, E.C. Andersen

**Affiliations:** 1 Department of Molecular Biosciences, Northwestern University, Evanston, IL; 2 Interdisciplinary Biological Sciences Graduate Program, Northwestern University, Evanston, IL; 3 Robert H. Lurie Comprehensive Cancer Center, Northwestern University, Chicago, IL; 4 Chemistry of Life Processes Institute, Northwestern University, Evanston, IL; 5 Northwestern Institute on Complex Systems, Northwestern University, Evanston, IL

**Keywords:** transposons, transposable elements, *C. elegans*, natural variation, QTL

## Abstract

Transposons are mobile DNA elements that generate both adaptive and deleterious phenotypic variation thereby driving genome evolution. For these reasons, genomes have mechanisms to regulate transposable element (TE) activity. Approximately 12–16% of the *Caenorhabditis elegans* genome is composed of TEs, of which the majority are likely inactive. However, most studies of TE activity have been conducted in the laboratory strain N2, which limits our knowledge of the effects of these mobile elements across natural populations. We analyzed the distribution and abundance of TEs in 208 wild *C. elegans* strains to better understand how transposons contribute to variation in natural populations. We identified 3,397 TEs as compared with the reference strain, of which 2,771 are novel insertions and 241 are TEs that have been excised in at least one wild strain. Likely because of their hypothesized deleterious effects, we find that TEs are found at low allele frequencies throughout the population, and we predict functional effects of TE insertions. The abundances of TEs reflect their activities, and these data allowed us to perform both genome-wide association mappings and rare variant correlations to reveal several candidate genes that impact TE regulation, including small regulatory piwi-interacting RNAs and chromatin factors. Because TE variation in natural populations could underlie phenotypic variation for organismal and behavioral traits, the transposons that we identified and their regulatory mechanisms can be used in future studies to explore the genomics of complex traits and evolutionary changes.

## Introduction

Transposons are mobile DNA sequences found in most eukaryotes and prokaryotes. Because these mobile elements can insert into genes to create loss-of-function phenotypes or move endogenous DNA sequences around genomes to create gain-of-function phenotypes, transposable elements (TEs) often contribute to genomic variation and evolution. For example, the increase of black-colored pepper moths during the Industrial Revolution resulted from selection for individuals with a transposon insertion into a cell-cycle regulatory gene that enabled rapid adaption to the highly polluted environment (van’t Hof et al. 2011). The movement of transposons can alter gene regulatory mechanisms (Girard and Freeling 1999; Slotkin and Martienssen 2007) and result in phenotypic variation by excising full or partial copies of neighboring genes and transporting these gene sequences to new locations in the genome (Moran 1999). Transposons can also insert into coding sequence (CDS) regions to produce loss-of-function alleles (Girard and Freeling 1999). Through these mechanisms, TEs can serve as sources of novel variation that can lead to phenotypic differences and act as important drivers of evolutionary change (Feschotte and Pritham 2007) by creating beneficial adaptations to new environments (Brookfield 2004; Naito et al. 2009; van’t Hof et al. 2011; Rey et al. 2016).

The abundance and effects of TEs vary substantially among and within species. Yeast strains differ widely in copy number and distribution of transposons, and variation in TY transposons represents the greatest amount of genetic variation after single nucleotide variants (SNVs) (Gabriel et al. 2006; Bleykasten-Grosshans and Neuvéglise 2011; Bleykasten-Grosshans et al. 2013). In *Drosophila melanogaster*, the observation of elevated germline mutation rates and sterility known as hybrid dysgenesis led to the discovery of active transposons known as P elements, and further studies on TEs suggest that most transposon insertions in both inbred lines and natural populations are rare (Kidwell et al. 1977; Lee and Langley 2010; Petrov et al. 2011; Cridland et al. 2013). Studies in *D. melanogaster* also suggest that transposons beneficially imitate the roles of tandem repeats by assisting with telomere protection and elongation (Levis et al. 1993). In humans, ∼44% of the genome is composed of TEs (Lander et al. 2001). Most of these transposons are retrotransposons and are thought to be inactive. However, several elements from two of the largest families, Alu and LINE, are linked to several human diseases, including hemophilia, Duchenne muscular dystrophy, and Apert syndrome (Bennett et al. 2004; Mills et al. 2007; Cordaux and Batzer 2009; Hancks and Kazazian 2012; Ayarpadikannan and Kim 2014).

Approximately 12–16% of the *Caenorhabditis elegans* genome is thought to be transposon sequences, of which the majority are cut-and-paste DNA transposons (Waterston 1998; Sijen and Plasterk 2003). Only a few DNA transposons exhibit activity under laboratory conditions (Fischer et al. 2003; Zagrobelny et al. 2004; Bessereau 2006). Most of the active DNA transposons belong to the Tc1/mariner superfamily (Eide and Anderson 1985; Plasterk et al. 1999). Studies predominantly focused on these families have identified several genes that silence transposon movement (Vastenhouw et al. 2003; Lee et al. 2012). Transposon silencing can occur through multiple mechanisms, including chromatin remodeling, DNA methylation, and RNA interference (Sijen and Plasterk 2003; Slotkin and Martienssen 2007; Lee et al. 2012). In *C. elegans*, Tc3 transposons are silenced by the piwi-interacting small RNAs (piRNAs) that form RNA-protein complexes with piwi proteins to target TEs by base-pairing interactions and degrade the mRNAs in the germline (Batista et al. 2008; Das et al. 2008). *C. elegans* has few copy-and-paste retrotransposons, but some are known to be active and impact the functions of genes, as exhibited by the well characterized CER1 retrotransposon (Palopoli et al. 2008). Active retrotransposition has not been observed under laboratory conditions, but insertion of CER1 into the *plg-1* gene in many wild strains results in loss of male copulatory plugging. Before the *plg-1* finding, a small number of TE insertions were investigated in a handful of wild *C. elegans* strains and hypothesized to be selectively neutral (Dolgin et al. 2008).

A better understanding of transposon variation in natural populations can provide insight into genome and trait evolution, as several hypotheses about the nature of selection against TE insertions have been proposed (Nuzhdin 1999). Transposon insertions could be deleterious if they disrupt genes (Finnegan 1992; McDonald et al. 1997), if they produce TE-specific proteins that are costly to protein production and impact genome integrity (Nuzhdin 1999), or if they cause ectopic recombination among TEs in dispersed genomic regions (Montgomery et al. 1987; Petrov et al. 2003). In addition, the distributions of newly inserted TEs can be correlated with SNV data using genome-wide association (GWA) studies to explore the genetic basis of transposon regulation in wild populations. In this study, we analyzed transposon copy number and distribution using whole-genome sequence data for 208 strains that belong to 152 unique genome-wide haplotypes. Transposons were more often located on arms of chromosomes than on centers, matching the expectations of background selection (Cutter and Payseur 2003; Rockman and Kruglyak 2009; Rockman et al. 2010; Andersen et al. 2012). The characterization of transposon families across the wild isolate population revealed several strains with particularly high transposon copy number in given families, possibly as a result of natural variation in transposon regulation. In addition, we identified novel potential regulatory mechanisms, including previously unreported complementary alignments between several piRNAs and transposon sequences. Variation in the insertion and excision locations of TEs could underlie differences across natural populations, so this resource will be invaluable to studies of quantitative traits. More broadly, we present a rich data set of TEs and regulatory mechanisms that can be exploited to better understand how these important mobile DNA elements impact genome and trait evolution.

## Results

### The Genome-Wide Distribution of Transposons Across the *C. elegans* Species

**Fig. 1.**
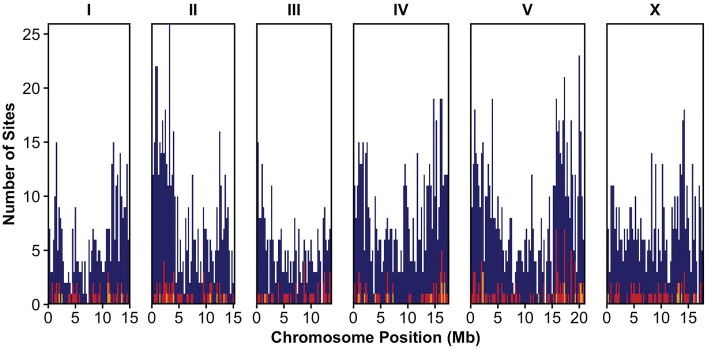
Distribution of transposon sites along the six *C. elegans* chromosomes. Counts represent the number of non-redundant transposon positions from all strains. DNA transposons are displayed in dark blue, retrotransposons in red, and transposons of unknown classification in yellow. Each bin represents 0.25 Mb. The *x*-axis ticks mark every 5 Mb.

Most of what is known about transposons in *C. elegans* has been learned through studies of the laboratory strain N2. The ∼100 Mb reference genome is annotated with 758 TE families, and only a few families of DNA transposons are thought to be active (Bessereau 2006). However, little is known about TE variation in natural populations (Dolgin et al. 2008). This variation may be controlled by genetic and/or environmental factors and can lead to large-effect alleles contributing to phenotypic variation. To better understand natural variation in transposon activities, we identified transposons using whole-genome sequence data from 208 wild isolate strains reduced to 152 unique genome-wide haplotypes or isotypes (Cook, Zdraljevic, Tanny, et al. 2016) using split-read data, sequences of transposon families, and annotated positions of transposons in the reference genome (see Methods section). We classified TE sites not present in the reference genome but present in a given strain (insertion sites), sites present in both the reference genome and a given strain (reference sites), and sites present in the reference genome but absent in a given strain (absence sites). Of the 224 transposon families annotated in Repbase (www.grinst.org/repbase) and Wormbase (www.wormbase.org), 184 were detected in the 152 strains. These transposon families consisted of DNA transposons, retrotransposons, and transposons with unknown classification distributed across the genome (fig. 1, table 1).

**Table 1:** Transposable elements sites can be classified by whether they are insertions, excisions of TEs as compared to the reference genome, or TEs sites shared with the reference genome. The 3,396 TEs can also be grouped into likely DNA elements, retroelements, or unknown TE types.

Total	3,396
Insertion sites	2,771
Active reference sites	241
Monomorphic reference sites	384
DNA elements	2,893
Retroelements	387
Unknown transposon elements	116

**Fig. 2.**
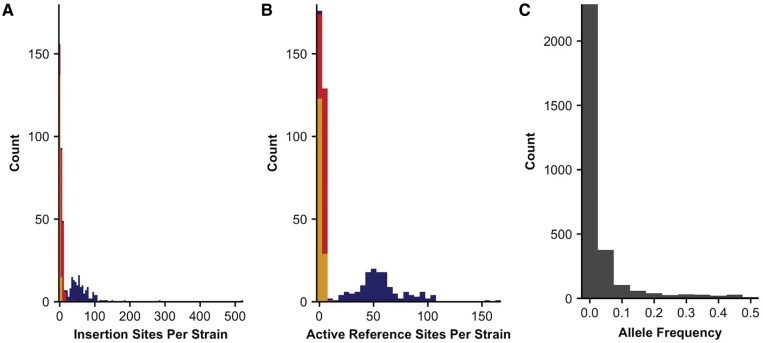
Distributions and frequency of transposon insertions and active reference sites. (*A*) Histogram of transposon insertions per strain with blue (DNA transposons), red (retrotransposons), and yellow (unknown transposons) bars shown. (*B*) Histogram of transposon excisions as compared with the reference genome with the same colors and classifications as in (*A*). (*C*) Minor allele frequencies are depicted for all positions at which an insertion site or active reference site was detected.

Out of the 758 TEs annotated in the reference genome, 131 TEs are annotated as Tc8, which we found to be too small to be detected reliably by the algorithms we used, and another TE was not called in our N2 strain or any wild strain. We considered the remaining 626 transposons further. Out of these remaining TEs, 385 (61.5%) sites were shared across all strains in the species, suggesting that these sites are inactive transposons or rarely move over long time scales (supplementary file S1, Supplementary Material online). We did not consider these sites further. The remaining 241 (38.5%) sites had at least one strain with a called absence, which implied that they could be active. We refer to the absence of a transposon at these positions as an “active reference” site in a given strain (supplementary fig. S1, Supplementary Material online). These active reference sites comprised DNA transposons (209, 86.7%), retrotransposons (17, 7.1%), and transposons of unknown classification (15, 6.2%) (table 1, supplementary file S2, Supplementary Material online). We also detected 2,771 unique insertion sites among all the strains (table 1, supplementary file S2, Supplementary Material online). Of these sites, 1,854 or nearly 67% were private to a particular strain (fig. 2), indicating activity of a transposon in a specific lineage. We did not find any insertion sites that were shared among all strains in the species implying that all of these TEs are likely active.

**Fig. 3.**
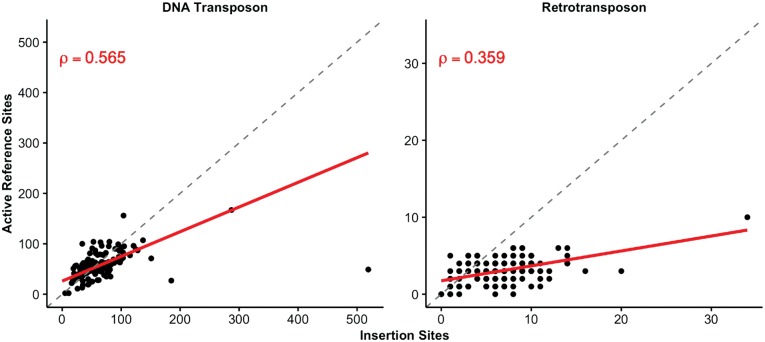
Active reference vs. insertion sites per strain. Each point represents an individual strain. The identity line is shown in dotted grey, and the line of best fit is shown in solid red.

As a proxy of transposon activity, we calculated correlations between total numbers of insertion and active reference sites per strain. The movement of “cut-and-paste” DNA transposons could lead to a high correlation between active reference sites and insertion sites if reference transposons are successfully excised and moved to a new location. In contrast, we expect that the correlation between insertion and active reference sites for “copy-and-paste” retrotransposons would be lower by comparison because strains would have an ever-increasing number of retroelements as they transpose. We separately compared the total number of insertion and active reference sites for DNA transposons and retrotransposons discovered per strain and found them both to be correlated (fig. 3). Similar levels of correlation were discovered when we compared the total number of these sites for DNA transposons and retrotransposons together (rho = 0.575, P = 8.92e−15) (supplementary fig. S2, Supplementary Material online).

The presence of an active reference site or an insertion site in a strain suggests transposon movement relative to the reference genome. We investigated the distribution of TE copy numbers per strain to assess the frequency of such activity. The average number of insertion sites per strain was 71.58 (range 5–523) (fig. 3; supplementary fig. S4, Supplementary Material online). The average number of active reference sites per strain was 61.32 (range 2–183). The spread of these distributions was wider for DNA transposons than for retrotransposons, which is consistent with previous suggestions that DNA transposons are more active than retrotransposons in *C. elegans* (Bessereau 2006).

Active TEs in *C. elegans* are sites of variation, like SNV sites, that can differentiate strains across a population. We investigated the relatedness of *C. elegans* strains when we account for SNVs and TEs separately or together. We expected that the TE distribution for each strain would be similar to the SNV distribution based on the low divergence among strains (Andersen et al. 2012). However, some discrepancies could occur because of the different mechanisms and time scales in which SNVs and TE transposition events are generated. We found that many strains that are related based on SNV data (Cook, Zdraljevic, Tanny, et al. 2016) are also related based on TE variation data (supplementary fig. S5, Supplementary Material online). For example, N2, the strain from which the reference genome was assembled, and LSJ1, a strain that shares a common ancestor with N2, both had the smallest number of insertion and active reference sites. Several strains that are divergent based on SNV data are also divergent based on transposon data, such as QX1211 (325 insertion sites and 183 active reference sites) and ECA36 (117 insertion sites and 168 active reference sites). However, some strains that are similar to the reference strain based on SNV data displayed considerable differences based on transposon data, such as strain ECA252 (186 insertion sites and 27 active reference sites). We also identified strains with a large number of TEs and confirmed existing strains known to have a large number of TEs. For example, CB4851, also known as the Bergerac strain, has the highest number of transposon insertions, 523, in the entire *C. elegans* population. Out of the 523 insertions in this strain, 406 belonged to the Tc1 family, whereas the average number of Tc1 insertions among all the strains was 7.14 (SD = ±35.18). Although CB4851 contained a high number of insertion calls, only 52 active reference sites were detected, suggesting that many transposons present in the reference genome are shared with CB4851. The similarities and differences between the relatedness of strains based on SNV and TE data can be used to investigate transposon regulation.

### Diverse Numbers and Distributions of TE Classes and Families

The variation in transposon copy number among the strains could be driven by differences in specific transposon families. Previous studies suggest that elements belonging to the Tc1/mariner superfamily are the most abundant throughout the *C. elegans* genome, and RNAi screens show that inhibition of certain genes can alter the transposition of Tc1, Tc3, Tc4, and Tc5 (Eide and Anderson 1985; Sijen and Plasterk 2003; Vastenhouw et al. 2003). We identified the transposon families that were present at the highest frequencies to determine the most prevalent families that might contribute the most to genotypic and phenotypic variation in natural populations. The most prevalent transposon could also be the most active in the genome. The DNA transposons with the highest number of total insertions among the strains, defined as those strains with TE counts greater than one standard deviation from the mean, included families Tc1, Tc3, Tc5, MARINER2, and MIRAGE1 (supplementary fig. S6 and table S1, Supplementary Material online). The Tc1, Tc3, and MARINER2 families also displayed the highest total active reference counts. Correlations between active reference sites and insertion sites for these families, assessed using Spearman’s rank correlation (rho = 0.44–0.65), suggested that these TEs are active. In addition, these results are concordant with previous studies showing high abundance of the Tc1/mariner superfamily. The retrotransposons with the highest number of total insertions included CELE45, LTRCER1, and CER2-I. These transposons could be active in natural populations, although retrotransposon activity has not been observed under laboratory conditions.

### Functional Consequences of Transposon Insertions

Active transposons can insert into genes to cause functional consequences. To determine if any genomic regions harbored large numbers of TEs, we divided the genome into 10-kb bins and counted TEs. Out of 9,980 regions, we found 481 that had a high number of TEs (top 5% of transposon counts), and out of these regions, 92 had three or more transposons. The 92 bins were more often found on arms than on centers (Chi-squared statistic = 21.381, P = 3.77e−06) and had more extreme values of Tajima’s D than regions with transposon counts in the bottom 95% according to a Wilcoxon rank sum test (P = 8.2e−03). If these TE insertions had functional consequences across the *C. elegans* species, we would expect to see strong evidence for selection in these bins over bins without a large number of TEs. However, only seven of these 92 regions had Tajima’s D values that would support evidence of selection (supplementary table S2, Supplementary Material online). We investigated the protein-coding genes disrupted by TE insertions within these seven genomic regions and found *C49G7.10*, *F39E9.6*, and *phat-3* with predicted deleterious insertion alleles. Each of these genes could be involved in interactions with environmental factors as an innate immune response gene, a negative regulator of small signaling molecules, and a pharynx gland cell expressed gene, respectively, suggesting that the signatures of selection that we observed could be mediating interactions in nature.

**Fig. 4.**
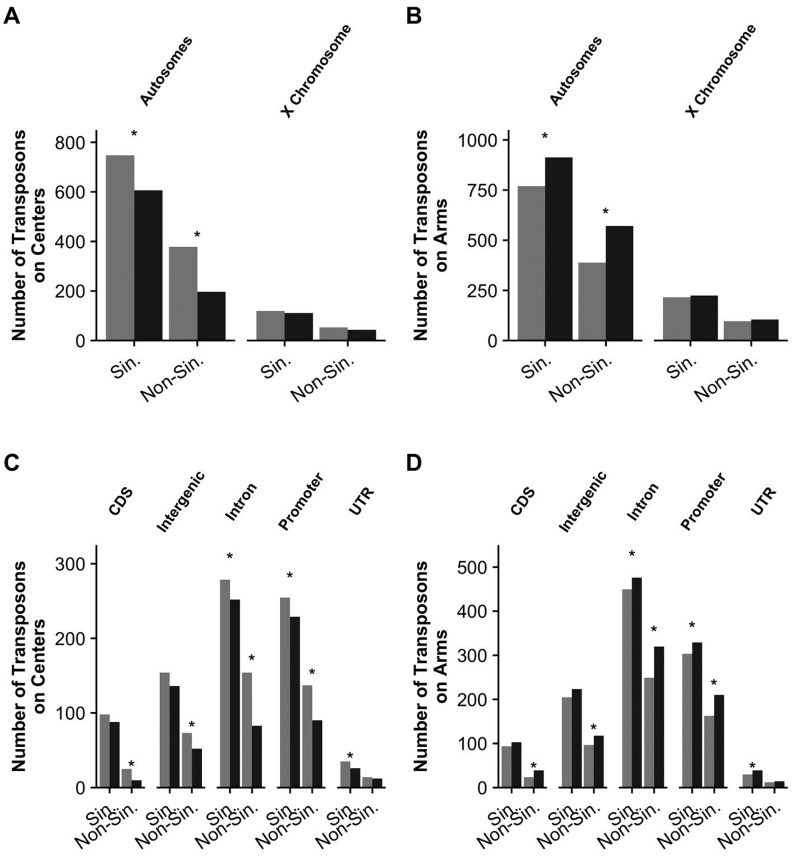
Numbers of observed and expected transposon insertions into different genomic regions. For each class of genomic region, the number of expected (gray) vs. observed (black) transposon insertions are shown. The expected number of transposon insertions were calculated based upon the size of the respective genomic classes. Arm and center chromosome estimates were summed from previous chromosome calculations (Rockman and Kruglyak 2008). TE insertions were classified as singletons found in one strain (“Sin.”) or non-singleton found in more than one strain (“Non-Sin.”). Chi-squared test statistics for the comparisons are found in supplementary table S3, Supplementary Material online. Stars denote significant deviations (*P* < 0.05) from expectations. Comparisons of TE insertions found on the centers (*A*) and arms (*B*) are shown. TE insertions into different genic classes (CDS, intergenic, intron, promoter, and UTR) are shown for centers (*C*) and arms (*D*).

The positions of transposons and their functional consequences can also be analyzed on a finer scale by investigating the types of genomic regions in which they are inserted. We determined whether or not transposons were more often found on chromosome arms (separated by autosomes or the X chromosome) than on centers and ran this analysis separately for TE insertions found in a single strain (singletons) or found in multiple strains (non-singletons) (fig. 4, statistics in supplementary table S3, Supplementary Material online). Regardless of allele frequency, more TE insertions were found on autosome arms than on centers (fig. 4*A* and *B*). This result suggests that like other variant classes TE insertions can accumulate on arms where their effects are less deleterious. We also investigated TE insertions into intergenic, CDS, promoter, and intron region categories (fig. 4*C* and *D*) separated into singleton and non-singleton insertions. Again, across all region categories analyzed, TE insertions were more prevalent on chromosome arms rather than centers. However, singleton TE insertions were more often found in introns, promoters, or UTRs rather than CDS or intergenic regions. The lack of enrichment of singleton TE insertions into intergenic regions on chromosome arms is unexpected but could reflect a signature of preventing ectopic recombination on chromosome arms (Petrov et al. 2003). In addition, the distributions of TE insertions were similar across the different types of TEs we analyzed (supplementary table S4, Supplementary Material online).

Transposons that are located in CDS regions or regions involved in gene regulation might have large fitness effects and therefore be selected against in wild populations. We found that 75.1% of unique TE insertions were located in protein-coding gene regions, 17.6% in intergenic regions, 5.0% in pseudogenes, and 2.2% in regions with other genomic classes. We further investigated the 2,253 insertion sites within protein-coding genes because variants in these regions are more likely to have functional consequences than insertions located in intergenic regions. Of these sites, 2,246 were unique to transposon type and were classified into the genic regions of promoter, 5′ or 3′ UTR, intron, or CDS (supplementary fig. S7, Supplementary Material online). Most of the insertions (50.4%, 1131) were located in introns where the detrimental effects of transposon insertion would be reduced. In addition, a large number of insertions were in promoter regions (34.9%, 784) or UTRs (4.1%, 91) where a transposon is less likely to insert into short gene-regulatory sequences. Only 10.7% (240) of the insertions were located in CDSs where they could affect the amino acid sequences of protein-coding genes.

Overall, most insertion sites were located in regions expected to have small effects, but several were located in regions that could cause mutant phenotypes. Insertions into essential genes would likely result in deleterious effects. Eighteen insertions were located in the CDS regions of essential genes with RNAi phenotypes that included “slow growth”, “sterile”, “embryonic lethal”, and “larval lethal” (supplementary table S6, Supplementary Material online), but the strains that harbor these insertions do not have reduced fitness (Cook, Zdraljevic, Tanny, et al. 2016). In addition, transposons were inserted into *cdr-2* and *vit-1*, which each exhibit easily visible mutant phenotypes when inhibited (Dong et al. 2005; Fischer et al. 2013). However, the wild strains with these insertions did not have the expected mutant phenotypes likely because both genes belong to gene families that can provide functional redundancy. Our data support the hypothesis that most transposon insertions do not have detrimental effects because either they are inserted into regions with little functional consequence or they are inserted into genes that belong to gene families that can provide redundant functions. Potential deleterious insertions into genes with uncharacterized phenotypic consequences remain possible, but future investigation is required to make these connections.

### Association Mapping of Transposon Abundances Across *C. elegans*

Our analyses suggest that the abundance and activity of transposons vary across the *C. elegans* wild isolate population. Some of this variation may be attributable to genetic factors. We used GWA analyses to identify genetic variation that impacts the regulation of transposon activities. Before mapping, we needed to define quantitative traits that represent TE activity, so we used the counts of transposons as proxies. These counts were calculated for insertion sites, active reference sites, or all TE sites. The TEMP algorithm calls TEs based upon alignment to known TE sequences (Zhuang et al. 2014), the so-called sites must have been recent events such that variation did not disrupt alignment. Therefore, we expect count traits can be correlated with genomic regions that contain variation in regulatory genes because TE activity is recent. We categorized transposons into three different trait groups: (1) total transposons, (2) transposons by type considered separately—DNA transposons, retrotransposons, or unclassified TEs, and (3) TE families considered separately—TEs grouped into 131 specific families by sequence similarity. These three groups could represent generalized mechanisms of transposon regulation (total TE counts), regulation mechanisms by the class of TE (type TE counts), or specific control mechanisms for each TE family (family TE counts). We calculated the TE counts of insertion sites, active references sites, and all sites for these three groupings. After filtering (see Methods section), 183 traits (3 total, 9 type, and 171 family) remained (supplementary table S7, Supplementary Material online). These TE count traits were mapped, and we detected 160 quantitative trait loci (QTL) for 87 traits (supplementary fig. S8, file S3 and S4 and table S8, Supplementary Material online).

**Fig. 5.**
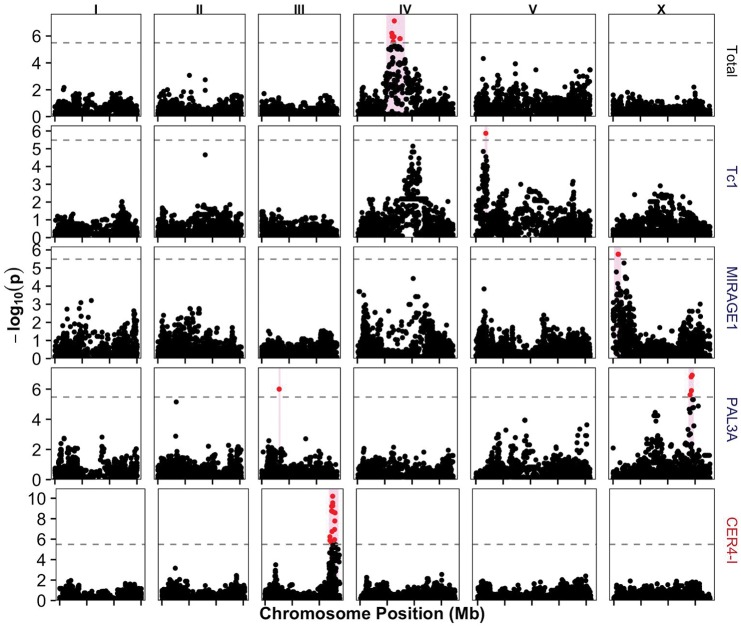
Manhattan plots for three TE count traits. These traits were filtered to retain QTL located more than 100 markers from positions with a high density of transposons and had good phenotypic splits. Total depicts the total number of insertion sites of any TE class. The mappings for Tc1 and CER4-I represent the transposon copy numbers of active reference sites. MIRAGE1 and PAL5A are the counts of insertion sites. The x-axis is the genomic position in megabase pairs (Mb) with the ticks marking every 5 Mb. The *y*-axis is statistical significance (−log_10_(*P*)). The significance threshold is indicated by the dotted gray line. SNVs that are above this threshold are shown in red, and SNVs that are below the threshold or do not pass our QTL filters (see Methods section) are shown in black. The genomic region containing 100 markers on either side of the peak marker are represented by the pink boxes.

For the three total TE count traits separated into insertion sites, active reference sites, and all TE sites, we identified the same QTL on chromosome IV for the count of insertion sites and all TE sites, but no QTL for the count of active reference sites (fig. 5, supplementary file S3, Supplementary Material online). For the nine transposon type count traits, we identified eight QTL (supplementary file S3, Supplementary Material online), but many QTL from insertion or active reference site count traits were shared with the total count traits. For the counts of DNA transposons, we identified two likely independent QTL—the counts of TE insertions, active reference sites, and all DNA transposons mapped to the same QTL on chromosome IV and the count of active reference sites trait had an additional QTL on chromosome II. The QTL on chromosome IV for DNA transposon insertion sites overlaps with the QTL for the total of all transposon sites and all transposon insertion sites, suggesting that the retrotransposons and unclassified TEs explain small amounts of the variation in the total TE traits. The QTL for total and DNA transposons on chromosome IV is not shared with any family-specific QTL mapping results and allowed us to investigate potential generalized regulation mechanisms of this transposon class. We tested the correlations of predicted functional variants (Cook, Zdraljevic, Roberts, et al. 2016) in the QTL genomic interval with the TE count data to identify candidate regulatory genes (supplementary table S9, Supplementary Material online). We found that none of the correlated variants were located in genes implicated in transposon regulation by previous studies (Vastenhouw et al. 2003; Lee et al. 2012), so we generated a list of novel candidate DNA transposon-regulatory genes. We identified natural variants that likely affect gene functions of *puf-11*, which encodes a putative RNA-binding protein, and two chromatin-regulatory factors—*hil-7*, which encodes a linker histone H1, and *cec-4*, which encodes a chromobox-containing protein. Future gene-specific experiments are required to define causal roles for these or other genes and variants in the regulation of DNA transposons. Variation in the counts of retrotransposons maps to two QTL for the number of active reference sites (chromosomes II and IV) but not for insertions or all sites. These QTL are distinct from the QTL identified for DNA transposon traits. Some TEs are not classified as DNA transposons or retrotransposons. The counts of these unclassified elements map to the chromosome II QTL shared with retrotransposons and a new QTL on the right of chromosome IV. These results suggest that some of these unclassified elements could be retrotransposons.

Natural variation in the abundance and distribution of specific families of transposons might be correlated with TE regulatory mechanisms that are different across the *C. elegans* species. For the count traits of TEs from families, we identified 150 QTL (supplementary files S3 and S4, Supplementary Material online). We found that ∼73% of significant QTL from family count traits were located within 100 markers of a site with a high density of transposons that belonged to the same family and type of site (*i.e*., insertion or active reference site) being mapped. These QTL might result from variation at the site of a TE or nearby its target site (*cis* variation). However, because we mapped sequence-based traits, the QTL could be driven by linkage disequilibrium between the transposon cis variation and the SNV marker used in the mapping, as has been observed previously for CER1 (Rockman and Kruglyak 2009; Andersen et al. 2012). We could not distinguish between these two possibilities, so we analyzed QTL found more than 100 markers from identified TE sites, which corresponds to a significant drop in linkage disequilibrium in most chromosomal regions (Rockman and Kruglyak 2009). Only 31 out of our 150 family-specific QTL remained after filtering (fig. 5, supplementary file S3, Supplementary Material online). We found overlaps between the type and family-specific QTL, suggesting that some of the type traits could be driven largely by abundant transposon families. The count of active reference DNA transposons mapped to a QTL on chromosome II that overlaps with QTL for active reference counts of Tc3, Tc5, and Tc6. All three of these family-specific QTL are likely driven by *cis* variation, which could drive the type DNA transposon trait as well. We also found that the overlapping QTL for active reference sites of retrotransposons and unclassified transposons on chromosome II overlaps with a QTL for the retrotransposon LINE2C active reference sites. The QTL for LINE2C, again, is likely driven by *cis* variation, suggesting that the type unclassified transposon trait might be related to this transposon family. The additional active reference transposon QTL for retrotransposons on the left of chromosome IV and unclassified transposons on the right of chromosome IV look to be correlated with QTL for the retrotransposon RTE1 and an unclassified (but likely retrotransposon) WBT00000617, respectively.

For the family-specific QTL not confounded by potential *cis* variation, we pursued fine-mapping approaches to identify putative transposon regulatory mechanisms. We measured correlations between the functional variants across our mapped genomic intervals and the counts of family-specific transposon sites (supplementary table S9, Supplementary Material online). The differences in the counts of Tc1 active reference sites correlated with variation in the gene *hil-6*, which encodes another linker histone H1, like we found for the TE type mappings of DNA transposons. In addition, we found this trait correlated with variation in the piRNA *21ur-9485*, although this piRNA does not align to the Tc1 consensus sequence. We found that the number of MIRAGE1 TE insertions correlated with variation in *nrde-3*, which encodes an Argonaute protein (Guang et al. 2008). This trait also correlated with predicted functional variation in *set-33* (Andersen and Horvitz 2007). Variation in the histone-encoding gene *his-72*, the exonuclease-encoding gene, *exo-1*, and a factor involved in P-granule function, *epg-3* were correlated with differences in active reference counts of CER4-I. Additional candidate genes with correlated variants among these traits have not been associated with transposon regulation previously, implicating novel factors in TE regulation.

### Potential Transposon Targets of piRNAs

In addition to the protein-coding genes that regulate transposon activities, the piwi-interacting small RNA genes (piRNAs) are also hypothesized to silence transposon movement (Weick and Miska 2014). Nucleotide variation in 21-nucleotide piRNAs might lead to altered binding to target RNAs and differences in the regulation of TEs. Because we only know of one piRNA-TE regulatory pairing (Batista et al. 2008; Das et al. 2008), much is still unknown about which piRNAs regulate transposon sequences. To investigate this question, we aligned all known piRNA sequences to our annotated collection of TE sequences using BLAST (Altschul et al. 1997) allowing for up to five mismatches in the alignment (supplementary tables S10 and S11, Supplementary Material online). Alignments with few mismatches might indicate that the piRNA could target and regulate transposon activities. At zero mismatches, ten pairings were discovered. The known piRNA-Tc3 pairing was not found because the two piRNAs that regulate Tc3 are not annotated in Wormbase. When we manually checked this pairing using our alignment scripts, we identified this interaction, validating the alignment methods for target identification. When more mismatches were allowed, additional pairings were discovered. However, we found that none of the identified QTL genomic intervals contained piRNAs that paired with the transposon that mapped to that region. These results suggest that either these small RNAs are not involved in TE regulation or that simple alignments are not sufficient to explain the regulation of TE activity.

### Rare Deleterious Variation in Transposon-Control Genes Is Correlated with Increased TE Copy Number

The GWA mapping experiments identified correlations between common genotypic variation shared among more than 5% of the strains across the *C. elegans* population. This approach will miss rare variants found in one or a small number of strains that might cause differences in the activities of TEs. For this reason, we identified strains with a transposon copy number at least four standards deviations away from the average for a particular TE count trait (supplementary table S12, Supplementary Material online). In these strains, we searched for variants in known TE-control genes (Vastenhouw et al. 2003; Lee et al. 2012) that were predicted to cause moderate to severe functional effects by SnpEff (Cingolani et al. 2012) and in piRNA genes. We uncovered 653 variants within the piRNA gene sequences in 21 strains that harbored extreme numbers of 56 different transposon family count traits (supplementary table S13, Supplementary Material online). In addition, this analysis identified 131 moderate or severe effect variants in TE control genes (supplementary table S14, Supplementary Material online). We used Grantham scores (Grantham 1974), a measure of evolutionary distance based on amino-acid polarity, composition, and molecular volume, to predict which of these variants might have the greatest phenotypic effects on the TE-control genes. The Grantham scores indicated that almost 93% of the non-synonymous variants predicted by SnpEff to have moderate or severe phenotypic effects are likely not strongly deleterious (conservative, 38%; moderately conservative, 44%; and moderately radical, 11%). Approximately 7% of the remaining variants (9 out of 131) predicted by SnpEff to have moderate or severe phenotypic effects also had radical changes by Grantham score (supplementary table S16, Supplementary Material online). For example, a cysteine-to-tyrosine variant in the gene *wago-1* is only found in the strain QX1791, which also has a large number of transposon insertions for HelitronNY1 and MARINER4. These rare deleterious variants are likely to affect the regulation of transposons and to generate phenotypic effects when these TEs insert into genes, providing us with a set of candidate variants that might play a role in phenotypic changes.

## Discussion

### Transposons Insert Preferentially into Gene-Poor Genomic Regions or into Genes with Functional Redundancy

In addition to activity levels, the locations in which transposons insert into the genome might contribute to the severity of their functional effects. We found that the majority of TE insertions were located in intergenic regions, promoters, or introns of genes found on chromosome arms. Chromosome arms are relatively gene poor and have higher recombination than chromosome centers (Rockman and Kruglyak 2009). This recombination profile is hypothesized to cause the increase in SNVs on chromosome arms relative to centers because of background selection (Cutter and Payseur 2003; Rockman et al. 2010). However, the three prevailing hypotheses about the nature of selection against *Drosophila* TE insertions (Nuzhdin 1999) would argue the opposite. Transposons should insert into regions of low recombination to limit ectopic recombination with homologous elements dispersed throughout that chromosome. Perhaps in *C. elegans*, ectopic recombination is less of an issue than gene disruption so inserts are often found in gene poor regions that have higher recombination. We did not observe any differences in the distributions of DNA transposons or retroelements, so similar mechanisms could explain the nature of selection on both classes of TE insertions.

Although transposons located in intergenic regions, promoters, or introns of genes might affect gene regulation, we focused on transposons that are located in CDS regions and can have detrimental functional consequences by disrupting CDSs. Such insertions are often lost through purifying selection and are rare (Rizzon et al. 2003; Quadrana et al. 2016). Likewise, we found few TE insertions into CDS regions, and of these insertions, even fewer were in the CDS regions of genes with lethal RNAi phenotypes in the laboratory strain background. In all of these cases, we found insertions into genes with presumptive redundant homologs or paralogs that would be knocked down by RNAi but not by a TE insertion into a single member of a gene family. In some cases, the gene with the TE insertion contained several missense mutations, indicating relaxed selection. By contrast, the related gene, providing the presumptive redundant function, did not have any variation across the species. These TE insertion data provide the raw material for many future experiments to identify functional consequences for transposons in the *C. elegans* species.

### Common and Rare Variation Underlie Differences in Transposon Activity across the Species

Counts of TEs in each strain were used as quantitative traits that represent activities of TEs across the species. We found that the majority of the 160 QTL and the locations of the TEs used to generate the count data overlapped. These results suggest that those QTL might be caused by variation at the site of a TE or by linkage disequilibrium between the presence of a TE and the SNV marker used in the mapping. We filtered these results to focus on a small number of QTL genomic intervals that likely contain regulators of specific TEs. The count of the total number of TEs and the count of the total number of TE insertions both map to a QTL on chromosome IV, which overlaps with a QTL just below the significance threshold for Tc1 insertion counts (fig. 5). Two genes that encode chromatin-regulatory proteins, *hil-7* (a H1 linker histone) and *cec-4* (a chromobox-containing factor), harbor correlated variation with TE counts underlying this QTL, suggesting this mechanism could regulate DNA transposons as a general class or the most abundant DNA transposon member, Tc1. In addition, the mapping of Tc1 counts identified a QTL on chromosome V, which also has a correlated candidate gene that encodes an H1 linker histone (*hil-6*). These two results suggest that linker histones might be a common mechanism to limit the spread of Tc1 and other DNA transposons. Variation in the number of MIRAGE1 TE insertions was correlated with variation in *nrde-3*, which encodes an Argonaute protein. NRDE-3 binds secondary small-interfering RNA in the cytoplasm and transports them to the nucleus where the complex associates with complementary nascent transcripts (Guang et al. 2008). It then interacts with NRDE-2 and NRDE-4 to stimulate histone 3 lysine 9 trimethylation, pause RNA polymerase II, and inhibit transcription (Burkhart et al. 2011). For the regulation of retrotransposons, we found that many of the variants correlated with the difference in CER4-I active reference sites were located in the histone-encoding gene *his-72*. Differences in the distribution and modification of histones have been shown to affect transposon silencing by affecting heterochromatic regions of the genome (Gendrel et al. 2002).

In addition to protein-coding genes, piRNAs have also been proposed as regulators of transposon movement (Lee et al. 2012; Weick and Miska 2014). Over 15,000 piRNA sequences have been discovered in *C. elegans*. Despite this large number, most piRNAs sequences are not complementary to that of transposons (Lee et al. 2012). We discovered ten transposons that share perfect complementarity with piRNA sequences based on BLAST alignments. These piRNAs potentially target the transposons to which they align. 151 additional alignments of piRNAs and transposons were identified when one to two mismatches were allowed, providing further evidence of specific transposon targeting. Variation that occurs in either a piRNA or transposon sequence may also disrupt the transposon-silencing mechanism or lead to differential transposon copy number in strains of *C. elegans*. However, we did not see any overlap between the piRNA-TE pairings identified by alignments and the piRNAs located in the QTL confidence intervals for these specific TEs. This result indicates that either the piRNA pairings we identified do not represent regulatory relationships or that piRNAs bind to target sequences independent of the complementarity we found. We require a better understanding of piRNA binding to target sequences to predict regulated target genes, including TEs. Because the mechanisms controlling the regulation of TE activity by piRNAs has remained elusive, these pairings between TEs and piRNAs provide data to address this important question.

### Transposon Abundance and Distributions Contribute to Understanding Evolutionary Processes and Complex Traits

Using whole-genome sequence data, we defined the number and distribution of transposons across 208 diverse *C. elegans* wild strains. The presence or absence of a TE is a genotypic marker that can be correlated with phenotypic differences. Therefore, this catalog gives the community the ability to investigate the genetic causes of quantitative traits using a unique marker set. Because in many species TEs underlie phenotypic differences important for evolutionary change, these data will facilitate studies of *C. elegans* adaptations in nature. In addition, we can probe this variation to make predictions for how TEs affect the functions of genes, so wild strains with TE insertions into specific genes provide mutant alleles for future studies. Given the power of *C. elegans* to map the genes that control quantitative traits (Reddy et al. 2009; Gaertner and Phillips 2010; Ghosh et al. 2012; Andersen et al. 2014; Noble et al. 2015; Cook, Zdraljevic, Tanny, et al. 2016; Large et al. 2016), these data will provide both the markers to help identify quantitative trait genes and the functional variants that could underlie complex traits.

## Materials and Methods

### Acquisition and Alignment of *C. elegans* Sequences

Whole-genome sequence data was obtained from 208 C. elegans strains as described previously (Cook, Zdraljevic, Tanny, et al. 2016). In brief, paired-end 100 bp sequencing was conducted using the Illumina HiSeq 2500 system, and sequences were demultiplexed and trimmed using the fastx barcode splitter tool of the FASTX-Toolkit v0.0.14 (http://hannonlab.cshl.edu/fastx_toolkit/) and Trimmomatic v0.32 (Bolger et al. 2014). Trimmed reads were aligned to the WS245 reference genome (http://www.wormbase.org) with BWA v0.7.8-r455 (Li and Durbin 2009). Strains with concordance greater than 99.93% were grouped together as the same genome-wide haplotype, as done previously (Andersen et al. 2012; Cook, Zdraljevic, Tanny, et al. 2016). This grouping left 152 unique strains in our analysis set (see supplemental file S1 in Cook, Zdraljevic, Roberts et al. 2016). All raw reads are deposited in the Sequence Read Archive with project number PRJNA318647. Alignment and variant call format (VCF) files are available from the *C. elegans* Natural Diversity Resource (CeNDR, http://www.elegansvariation.org/Data) (Cook, Zdraljevic, Roberts, et al. 2016).

### Sources of Transposon Sequences

The ability to identify transposons and quantify transposon copy number often relies on a well curated set of transposon consensus sequences and annotated positions of transposons in the reference genome. Consensus sequences of 152 transposons were obtained from Repbase (January 13, 2015). In addition, full and partial copies of TE sequences were extracted from the *C. elegans* WS245 reference genome (http://www.wormbase.org). Input sequences for the transposon-detection programs consisted of all sequences obtained from Repbase and WormBase, but final outputs are reported according to family annotation. Out of 758 TEs available on WormBase, 593 were annotated with a transposon family. The names of TEs from WormBase are shortened from “WBTransposon” to “WBT” in all figures and tables. To avoid redundancy in family names between the two databases, TEs from WormBase were compared to the Repbase family consensus sequences using blastn searches (Altschul et al. 1997). TEs grouped as a family in WormBase were reassigned a Repbase family name if the majority of elements had top BLAST hits to a Repbase consensus family and at least one of the elements had an E-value of zero when compared with the Repbase family. TEs without an assigned family name in WormBase were classified with the Repbase family name of the top BLAST hit, if the E-value was zero. All TEs without a match in the Repbase database were compared with each other with blastn, and an element was grouped into a family with any other element to which it had a BLAST result with an E-value of zero. The positions of transposons in the reference genome were obtained from WormBase, and the position of a known CER1 transposon was manually added at III:8852596–8861460 (Palopoli et al. 2008) (supplementary table S15, Supplementary Material online). The ability of the transposon-detection programs to detect elements of a short length is reduced, so we analyzed transposon families in which a large proportion of the elements belonging to that family were greater than 100 bp in length, which eliminated the Tc8 TE family from our analyses. After this restriction, information from WormBase was available for 627 TEs. One of these TEs was not found in our N2 genome or in any wild isolate, so it was removed leaving 626 TEs. After blastn comparisons, the transposons from WormBase and Repbase could be classified into 224 unique families (supplementary table S16, Supplementary Material online, and FASTA at www.andersenlab.org/Data/Laricchia_etal).

### Simulations

The performances of three transposon-detection programs, TEMP (Zhuang et al. 2014), RetroSeq (Keane et al. 2013), and TE-Locate (Platzer et al. 2012) were evaluated based on their abilities to detect transposon insertions. These programs were chosen because they each accept BAM/SAM files as inputs and support BWA-MEM alignments. The mismatch and minimum score difference parameters in TEMP were set to 1 and 30, respectively. For calls detected by TEMP, only transposons with reads supporting the call at both ends were retained. The minimal distance to count, minimal support reads, and minimal supporting individuals parameters in TE-Locate were set to 1000, 3, and 1, respectively. RetroSeq was run with the optional ‘align’ parameter set to true and the minimum percent ID set to 0.70. The ability of these programs to detect new insertions of transposons relative to the reference genome was assessed by running the programs after using BamSurgeon (github.com/adamewing/bamsurgeon) to simulate 1,000 sequences of TEs in an N2 BAM file in regions without pre-existing transposons and without excessive coverage (>1,000× coverage). BamSurgeon successfully inserted transposons at ∼70% of the 1,000 sites chosen for simulations to create a set of eight BAM files containing different TE insertion distributions.

To remove redundant calls, which may occur if two or more transposon elements are classified in the same family and consist of highly similar sequences, adjacent transposons detected by the insertion callers were collapsed into one transposon if they belonged to the same family and were separated by a length less than the longest element sequence from that of the corresponding family. The transposon with the highest read support was used to determine the base pair location of the collapsed transposon. The distance between the start position of a simulated transposon and the start position of the nearest detected transposon was calculated with the closestBed tool within the BEDtools v2.17.0 suite (Quinlan and Hall 2010). Only transposons annotated with the same family as the nearest simulated transposon within varying designated cutoff distances were considered true positives. The average true positive rate (TPR) and false discovery rate (FDR) were calculated after running the transposon–detection programs on eight rounds of simulations across cutoff distances ranging from 1 to 50 bp (supplementary fig. S9, Supplementary Material online). By analyzing the TPR and FDR using different program parameters, TEMP was chosen to detect insertions and absences of transposons relative to the reference genome. As an initial assessment of accuracy, we required a detected transposon insertion to be located at the exact base pair location (where it was simulated) to be considered a true positive. Using this strict criterion, the TPR and FDR among the four simulated levels of coverage ranged from 69.23% to 90.04% and from 23.62% to 16.86%, respectively (supplementary fig. S10, Supplementary Material online). To further assess sensitivity, the TEMP insertion caller was evaluated across various read support and population frequency thresholds irrespective of the distance between the simulated and detected transposon (supplementary figs. S11 and S12, Supplementary Material online).

The TEMP package also includes an absence caller that can be used to detect whether a transposon present in the reference genome is absent in a strain of interest. BamSurgeon could not be used to evaluate the performance of this caller because it does not allow one to specify the exact positions at which large structural variants should be excised, so TEs were instead inserted into the N2 reference genome. Unaltered N2 reads were realigned to this genome to test if TEMP would detect excisions at the locations where transposons were simulated in the reference genome but not simulated in the BAM file. One hundred TEs were inserted into regions of the reference genome without pre-existing transposons using RSVSim (Bartenhagen and Dugas 2013), and the performance of TEMP was evaluated after eight rounds of simulations. TE-Locate was also run on this set of simulations to evaluate its ability to correctly identify the maintenance of reference transposons in the strain of interest. The location of a reference transposon detected by TE-Locate can be reported several base pairs away from the actual position in the reference genome. To keep calls of reference transposons consistent among all samples, the closestBed tool in BEDtools was used to retain calls within 1 kb of an annotated reference transposon of the same family, and the positions of the transposons in the reference genome were kept as the base coordinates. For both the TEMP absence caller and TE-Locate reference caller, transposon calls were filtered to retain only those calls with read support above a minimum threshold. The TPR and FDR were calculated across a minimum read support threshold ranging from one to 30 reads (supplementary figs. S13 and S14, Supplementary Material online).

Accurately calling transposons requires sufficient sequence coverage in the strains. To determine how the TPR and FDR would change with varying coverage levels, the N2 BAM file was down-sampled to 50%, 25%, and 10% of the original depth of coverage with Picard Tools v1.94 (https://github.com/broadinstitute/picard). Simulations for the insertion, reference, and absence callers were then conducted using these new coverage levels. All program parameters and filters were chosen to reduce the amount of false discoveries without sacrificing many true positive calls. For the TEMP insertion caller, all calls were filtered to remove those calls that did not have a population frequency greater than 0.25 and at least eight reads supporting the presence of the transposon. For the TE-Locate reference caller and TEMP absence caller, all calls that did not have at least three reads supporting the call were removed. Based on eight simulations with the chosen parameters, a strain with a depth of coverage of 65× has a TPR of 97.23 for the insertion caller, 92.72 for the reference caller, and 94.0 for the absence caller. The FDR under such conditions is 2.24 for the insertion caller, 0.06 for the reference caller, and 1.95 for the absence caller. The TPR at 13× depth of coverage is 78.47 for the insertion caller, 56.16 for the reference caller, and 80.13 for the absence caller. The FDR at this coverage is 0.72 for the insertion caller, 0 for the reference caller, and 0.32 for the absence caller.

Performance was further evaluated by calculating the number of true positives and false positives removed after enforcing the filters. At 65× depth of coverage, applying the filters for the TEMP insertion caller removed 1.16% of true positive calls and 63.13% of false positive calls, and at 13× depth of coverage, 8.47% of true positive and 67.44% of false positive calls were filtered from the results (supplementary fig. S15, Supplementary Material online). For the TE-Locate reference caller, applying the read support filter resulted in the removal of 3.98% true positive calls and 72.73% false positive calls at 65× depth of coverage, and 34.58% true positive calls and 100.00% false positive calls at 13× depth of coverage (supplementary fig. S16, Supplementary Material online). After applying the filter for the TEMP absence caller, 0.92% of true positive calls and 80.52% of false positive calls were removed based on a simulated strain at 65× depth of coverage, whereas 9.21% true positives and 90% false positives were removed at 13× depth of coverage (supplementary fig. S17, Supplementary Material online). All strains investigated for this study have a depth of coverage above the lowest simulated coverage level of 13×. The maximum depth of coverage is 541.45× and the minimum depth of coverage is 21.11×. The median depth of coverage among all the strains is 69.53×, and the average depth of coverage is 89.27× (SD = ±65.43×).

### Calling Transposons in Wild Isolates

TEMP was used to detect insertions and absences of transposons relative to the reference genome in 152 wild strains. Scripts for transposon detection and analysis are available at https://github.com/AndersenLab/Transposons2 and https://github.com/AndersenLab/TransposonFigures. For the insertion caller, one mismatch was allowed when mapping a read to the consensus sequence, and reads were considered uniquely mapped if the score difference between the best and second best hit was greater than 30. The maintenance of reference transposons was determined using TE-Locate with the minimal distance to count, minimal supporting reads, and minimal supporting individuals parameters set to 1,000, 3, and 1, respectively. TEMP was used to detect absences of transposons. Transposon calls were processed as done for the simulations to collapse adjacent insertion calls and adjust positions of the reference calls. Only insertion calls with support at both ends of the insertion site, at least three reads of support, and a population frequency greater than 0.25 were maintained. Reference and absence calls with support from at least three reads were maintained. The TEMP absence caller occasionally defines one deletion that actually spans multiple transposons. In such cases, BEDtools was used to find which transposons were located within the span of such a deletion, and the absence call was split into multiple calls as necessary. All outputs from the transposons callers were filtered to remove multiple calls at the same position, maintaining only those calls with the highest read support. Only the start positions of transposons were reported in the results. If both a reference and absence call were made at the same position for a particular transposon in a strain, resulting in contradiction, the call with higher amount of read support was retained. The absence call made by TEMP was retained if the read support from both callers was equal. Among all 152 strains, 4,256 contradictory calls were detected out of the 86,253 total sites in which a reference or absence call was made (supplementary fig. S18, Supplementary Material online). To determine if removing the call with lower read support was an appropriate filter, calls for CER1, a transposon that has been studied using multiple detection methods in different strains of *C. elegans*, were analyzed and compared with previous results (Andersen et al. 2012). In this previous study, the presence or absence of CER1 in 202 strains was determined using PCR. Of the 152 strains in which transposons were called, 96 could be compared with the previous results. The calls for CER1 were in agreement for 93 out of these 96 strains. Sixty-four out of the 152 strains possessed contradictory calls from TEMP and TE-Locate at the CER1 site present in the reference genome. Maintaining only the call with higher read support resulted in 39 true positive calls and 2 false positive calls (supplementary fig. S19, Supplementary Material online). The validity of the remaining contradictory calls could not be determined because they belonged to strains not analyzed in the previous data set. The high TPR and low FDR provided support for the implemented method of resolving contradictory calls. Overall, the transposon–detection programs allowed us to detect the loss or maintenance of reference transposons in 152 wild strains as well as sites of transposon insertions. Each strain could then be scored across all the unique transposon sites.

A list of unique transposon locations was compiled from all the strains. If multiple strains had an insertion call within 50 bp of a transposon insertion of the same family in another strain, the calls were merged into one call, and the average base pair position among all the strains with that transposon was assigned as the insertion location. Each strain was scored for the presence or absence of a transposon at all unique transposon locations. If a strain had less than 8× coverage in the interval flanking an insertion location by 25 bp on either side, the strain was scored as “NA”. A strain was also scored as “NA” if it lacked a call by both the TE-Locate reference caller and the TEMP absence caller at locations of annotated transposons in the reference genome. In supplemental files S1 and S2, Supplementary Material online, marker positions are followed by “_NR” for insertion sites and “_R” for reference sites. All reference sites without at least one absence call in a strain and one reference call in a strain were removed. Transposon copy number per strain was measured as the total separate counts for insertion calls and absence calls. These total calls per strain were also calculated for each transposon family and for DNA transposons, retrotransposons, and transposons of unknown classification. The correlation between total numbers of insertion and absence, and reference and absence sites per strain was analyzed with a Spearman’s rank correlation test. A robust regression model with an MM estimation (Yohai 1987) was used to find the line of best fit. The total number of absence and reference calls were expected to be inversely proportional because the presence of one of these events for a specific transposon requires that the other event is absent or lacks enough coverage to detect. Absence and reference sites per strain were correlated (rho = −0.791, *P* = 2.2e−16) (supplementary figs. S20 and S21, Supplementary Material online). The information provided by these type of sites is redundant, and we therefore considered only absence sites in analyses, which we refer to as “active reference” sites.

### Annotating Genomic Features of Transposon Sites

The distribution of insertion sites was further analyzed based on the genomic features of their locations. Positions of coding regions, 5′ untranslated regions, 3′ untranslated regions, and introns were downloaded from WormBase (WS245). Promoter regions were defined as the regions 2,000 bp upstream of the start of genes or the region until the nearest gene, if such gene was less than 2,000 base pairs away. The window tool within the BEDtools suite was utilized to determine the genomic features in which transposon insertions were located. A transposon can be located in multiple genomic features if the base pair position of the insertion constitutes multiple isoforms (e.g., promoter of one gene and intergenic region of another gene). Therefore, only one splice-form annotation was recorded according to the following priority order: CDS, UTR, promoter, intron, and intergenic. Biotype information from WormBase, which denotes gene and transcript classification, was also recorded for transposons located in a gene. Biotypes were defined as “protein coding”, “pseudogene”, or “other”. The classification of “other” includes small RNAs, such as a snoRNA. Biotypes classified as NA were transposon insertions into intergenic regions. Out of the 2,771 transposon insertions, we classified 3,000 unique insertions into genomic features (supplementary table S5, Supplementary Material online). RNAi phenotypes of essential genes were obtained from WormBase, and transposon insertion sites were annotated with the RNAi phenotype if they were located in genes with phenotypes that include the keywords “arrest”, “lethal”, “sterile”, “slow growth”, “sick”, and “severe”. Insertions into several genes were investigated with the Integrative Genomics Viewer to confirm the presence of split and discordantly mapped reads that indicate the presence of a transposon insertion. Gene ontologies from WormBase were also used to annotate transposon insertion sites. Information on whether any transposon site was located on a chromosome arm or center was recorded based on breakpoints defined previously (Rockman and Kruglyak 2009), and a chi-squared test was used to establish whether more transposons were found on arms or centers with normalization for the number of base pairs found in each genomic region. This test and normalization were also applied for transposons locations in CDS, intergenic, and intronic regions. We also calculated the number of transposon insertions for each non-overlapping 10 kb segment of the genome to identify regions with a particularly high numbers of transposons, defined as bins with transposon counts in the 95th percentile or above for counts of TEs. We also assessed whether evidence for selection exists in any of these bins using Tajima’s *D* (Tajima 1989).

### Association Mapping

The association between transposon copy number and genetic variation was analyzed using GWA studies. These studies can be sensitive to missing data when evaluating count data of a sequence-based trait, such as transposon copy number. To avoid confounding the results, any transposon insertion positions that were scored as “NA” in 10% or greater of the strains were removed from the analyses. This filter eliminated 306 out of 2,771 TE insertions and 62 out of 241 active reference TEs. For a given trait, strains were removed from the analysis if they contained “NA” scores at greater than or equal to 10% of either insertion or active reference the sites. Many transposons did not have insertions or active reference sites, so zero TE count traits across all strains were eliminated. In addition, the count traits were filtered to remove traits in which fewer than 5% of the strains differed in the distribution of a TE count to reduce the effects of outliers.

GWA study outliers were eliminated from raw transposon calls using the BAMFprune function (Binned Anomaly Mitigate and Fit) in the “easysorter” package available at https://github.com/AndersenLab/easysorter. In brief, outlier strains with phenotype values outside of the population distribution were eliminated if they comprised less than 5% of the population. Mappings were conducted using the gwas function in the rrBLUP package (Endelman 2011). The input kinship matrix was generated using imputed whole-genome SNV information produced using the rrBLUP package and consisted of SNVs identified by Andersen et al. (2012) using RAD-seq and whole-genome SNV data (Cook, Zdraljevic, Tanny, et al. 2016). Only SNVs with a minimum of 5% minor allele frequency were used in the mappings. A set of RAD-seq SNV calls that could distinguish between all haplotypes among the strains was lifted over from WS210 to WS245 and pulled from the whole-genome data set for the 152 strains tested in this study. Ranges for genomic regions of interest were determined by simulation, and for a given trait, the region of interest was found to be within 50 variants upstream or downstream from the last variant above the Bonferroni corrected *P*-value threshold. SNVs above the Bonferroni threshold were grouped into one QTL peak if they were located less than 300 SNVs away from each other. Correlations between traits and variants from the whole-genome sequencing set within a given region of interest were measured using a Spearman’s rank correlation test. Only variants that had a moderate to severe predicted effect as determined by SnpEff (Cingolani et al. 2012) were used for these calculations. Absolute values of the Spearman’s rank correlation coefficient greater than the 90th quantile of all calculated coefficients were reported. Additionally, we filtered the QTL mapping results to remove QTL where the median TE count values (when split by the QTL peak marker genotype) were the same because many of our TE count traits were bimodal. This filter removed 11% of the QTL for counts of family TEs, which could be caused by allele-frequency skews from outlier effects or represent the presence of background-specific modifiers. The mapping algorithm was run for several measurements of transposon copy number. For every category, we counted the total number of TE sites, the number of TE insertion sites, and the number of active reference sites. The different categories were (1) total TEs, (2) total TEs divided into DNA transposons, retrotransposons, and transposons of unknown classification, and (3) total TEs divided into the 131 TE families.

Mapping a genomic trait, such as transposon copy number, often identifies QTL at the positions of the transposons in the genome as a result of either local variation and mapping to the transposons themselves. To avoid confounding our results by examining QTL mapping to the transposons themselves, GWA mapping results were screened to identify the traits that contained QTL that were not located near sites with high transposon counts for a particular family and were not created by minor alleles enriched in rare strains in the population. The cegwas (https://github.com/AndersenLab/cegwas) package was then used to measure linkage disequilibrium between QTL and examine which genes within the region of interest contained SNVs within protein-coding regions that were correlated with the phenotype. In addition, positions of piRNAs were obtained from WormBase, and the QTL were examined to determine if the regions of interest overlapped any piRNAs with variation across the strain set.

### Examining Factors of Transposon Control

Regulation of transposon movement has also been linked to piRNAs, although complementary piRNA sequences have been only described for one transposon, Tc3, in *C. elegans* (Batista et al. 2008; Das et al. 2008). To identify additional piRNAs that may complement and regulate transposons, all sequences of piRNAs were aligned to the transposon sequences used as input for the detection programs with BLAST using a word size of five and a maximum *E*-value of 0.1. Zero to five mismatches were allowed in the alignment. BLAST aligned the two known piRNA sequences that target the Tc3 transposon to the corresponding transposon sequence with zero allowed mismatches, demonstrating the utility of these searches.

GWA studies may not reveal QTL for traits in which only one or few strains are outliers. These strains were defined as those with a transposon copy number at least four standard deviations greater than average for insertion and active reference calls. Traits with only a single transposon site were excluded from this analysis as the four standard deviation cutoff is less informative. To determine if rare variants in transposon-control genes potentially affected the difference in transposon copy number among such strains, SnpEff was run on a list of genes found in previously published research to have an impact on piRNAs and transposon movement, as well as all mutator genes and piRNAs, which have been implemented in transposon control. An allele had to be present in 10% or less of the 152 strains to be considered a rare variant.

In addition to examining traits for outliers in transposon copy number, the positions of transposons were inspected to ascertain if any insertions are located in the CDS regions of genes that may result in lethal or easily visible phenotypes. These genes and their homologs were analyzed with SnpEff to determine if the homolog(s) lacked mutations of severe functional consequence(s), allowing it to recapitulate the function of the gene containing the transposon insertion. Genes that belong to a large gene family or have many close homologs were not analyzed because we assumed that at least one member of the family could functionally substitute for a rare transposon insertion into another gene in that family.

## Data Access

Raw Illumina sequence data are deposited in the Short Read Archive with project number PRJNA318647. Alignment and variant call format (VCF) files are available from the Caenorhabditis Natural Diversity Resource (CeNDR) Data page http://www.elegansvariation.org/Data. The locations of transposon insertions (at the position of the first base pair) can also be found on the variant browser on CeNDR. Because of file size restrictions, supplementary data and figures can be found at www.andersenlab.org/Research/Data.

## Supplementary Material

Supplementary data are available at *Molecular Biology and Evolution* online.

## Supplementary Material

Supplementary Data
