# Supplementary material for: Natural Variation in the Distribution and Abundance of Transposable Elements Across the Caenorhabditis elegans Species
Source: Mol Biol Evol. 2017 May 9;34(9):2187–202. doi: 10.1093/molbev/msx155 (PMC5850821; doi:10.1093/molbev/msx155)
Supplement: Supplementary Data [file msx155_Supp.zip › Laricchia_etal_SupplementalFigures.pdf]

SUPPLEMENTAL MATERIAL TABLE OF CONTENTS

Supplemental File S1. Presence/absence data for all positions at which a transposon was detected in at least one strain

Supplemental File S2. Presence/absence data for all non-monomorphic positions at which a transposon was detected in at least one strain

Supplemental File S3. PDF of all GWA mapping results, including genetic architectures, linkage disequilibrium between QTL, and genotype-by-phenotype splits.

Supplemental File S4. GWAS results for all traits mapped

Supplemental Table S1. Most prevalent transposons.

Supplemental Table S2. Tajima's D and transposon number of 10 kb regions with at least three transposons

Supplemental Table S3. Chi-square test results comparing distributions of transposons in different genomic regions

Supplemental Table S4. Chi-square test results comparing distributions of transposons on chromosome arms and centers

Supplemental Table S5. Genomic regions of transposon insertion sites

Supplemental Table S6. Transposon insertions in genes with lethal RNAi or easily visible phenotypes

Supplemental Table S7. Transposon traits used for GWAS analysis

Supplemental Table S9. Peak information for mapped QTL

Supplemental Table S10. Correlated variants within QTL

Supplemental Table S11. Counts of unique piRNAs and transposons from BLAST alignments

Supplemental Table S12. Combinations of piRNAs and transposons from BLAST alignments

Supplemental Table S13. Strains with extreme transposon copy numbers

Supplemental Table S14. Variation found in piRNAs

Supplemental Table S15. Variation found in protein-coding genes and Grantham scores

Supplemental Table S16. Positions of transposable elements in the reference genome

Supplemental Table S17. Sequences of transposable elements and family groupings

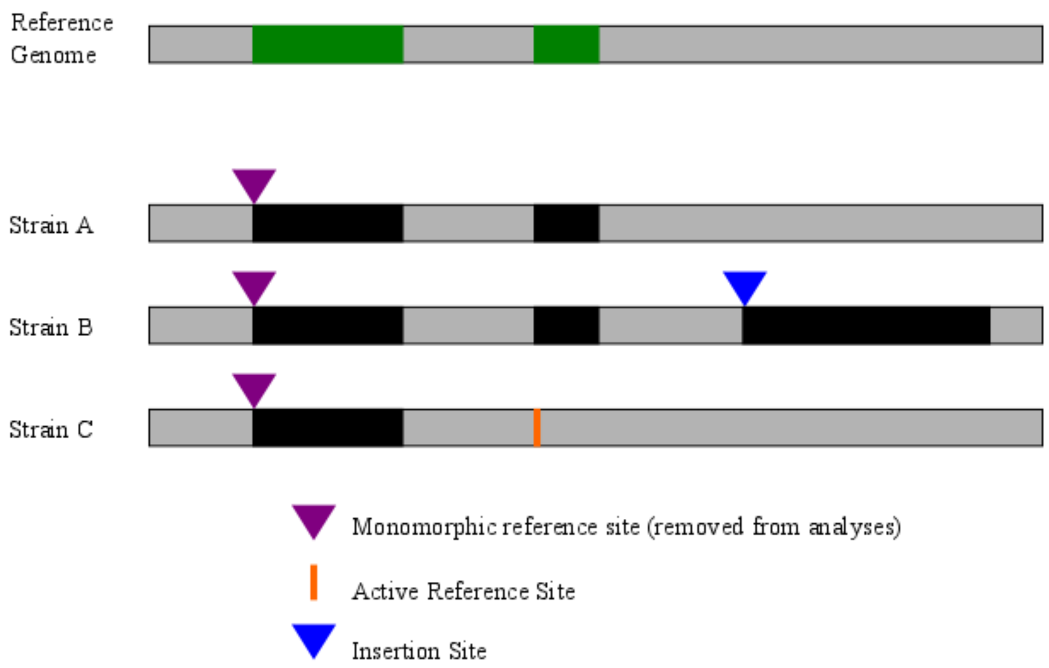

Supplemental Figure S1. Classification of transposon sites. In this example, the reference genome has two transposon sites (black rectangles). All three wild strains share one of the reference transposon sites (purple triangles). This monomorphic site was removed from our analyses. Strain B has a transposon insertion not found in the reference (blue triangle), and strain C has a transposon that has been excised and moved as compared to the reference (orange bar) denoting an active reference site.

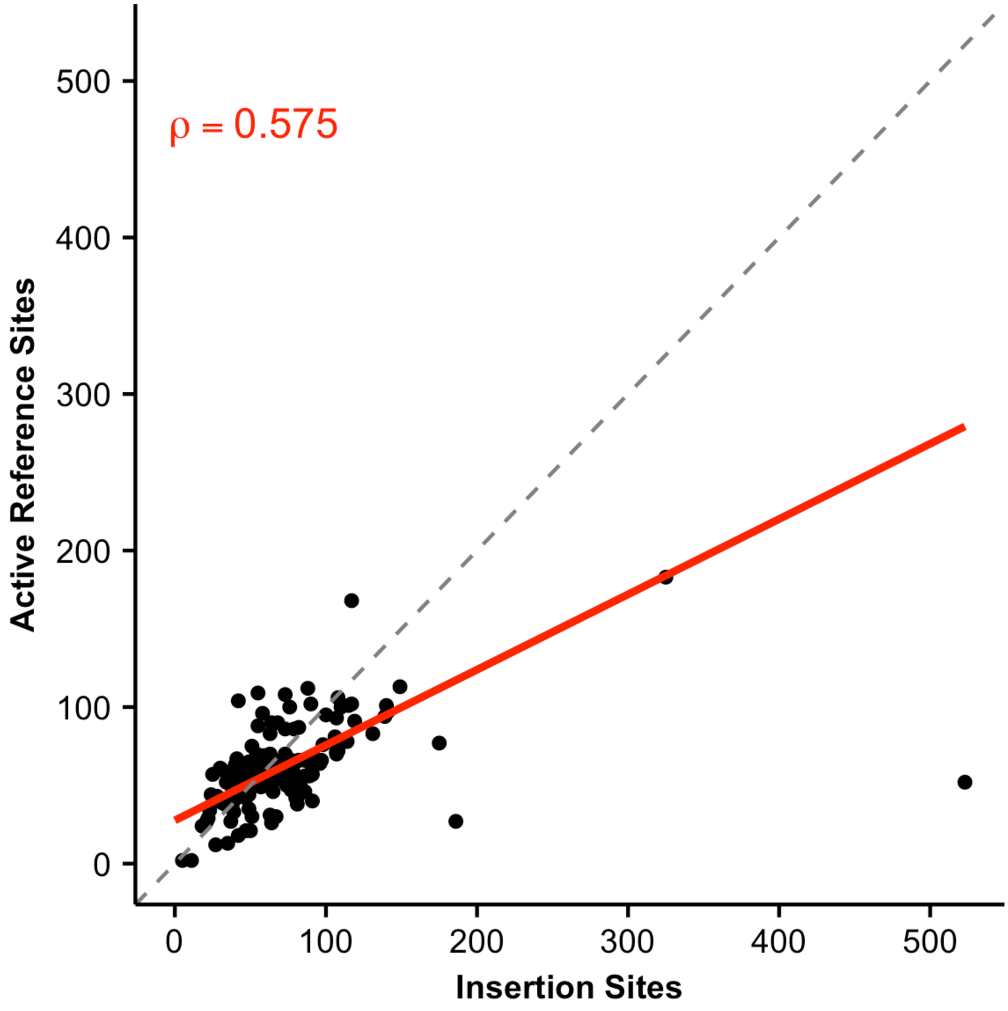

Supplemental Figure S2. Insertion sites vs active reference sites per strain. The number of insertion sites in a strain versus the number of active reference sites is plotted for each of the 152 strains. The identity line is shown in dotted grey, and the line of best fit is shown in solid red.

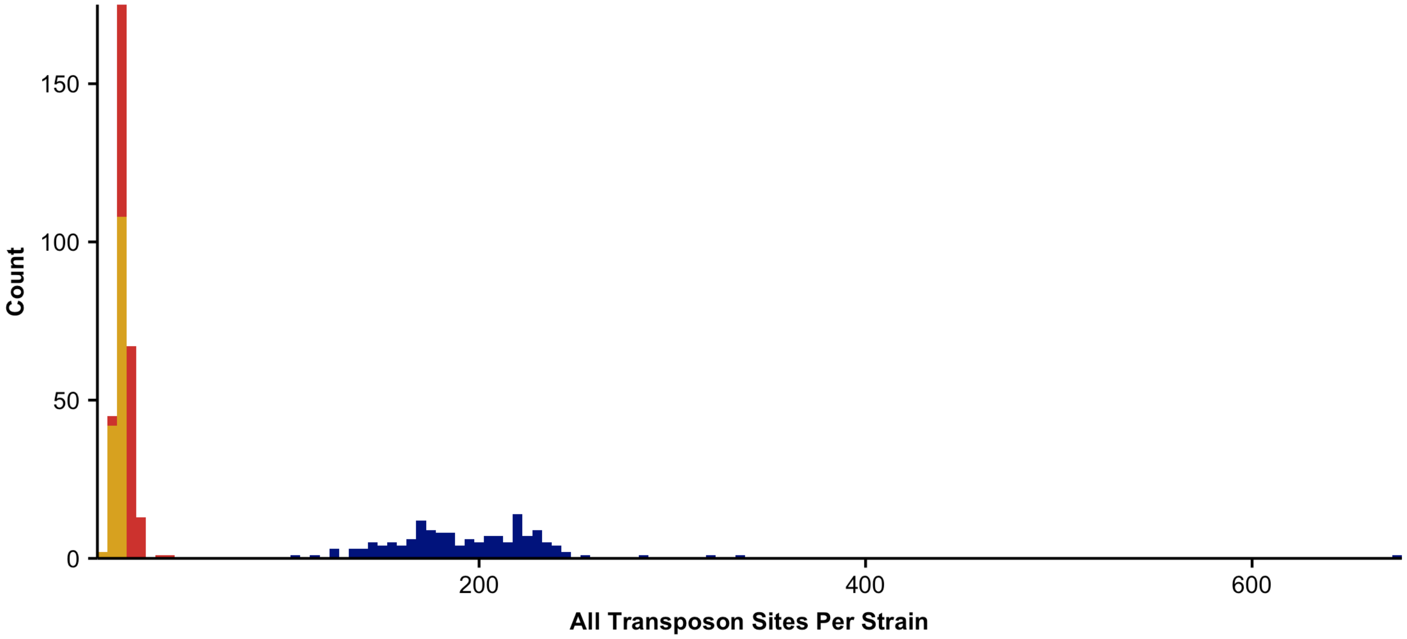

Supplemental Figure S3. Histogram of transposons per strain. Blue, red, and yellow bars denote DNA transposons, retrotransposons, and transposons of unknown classification, respectively.

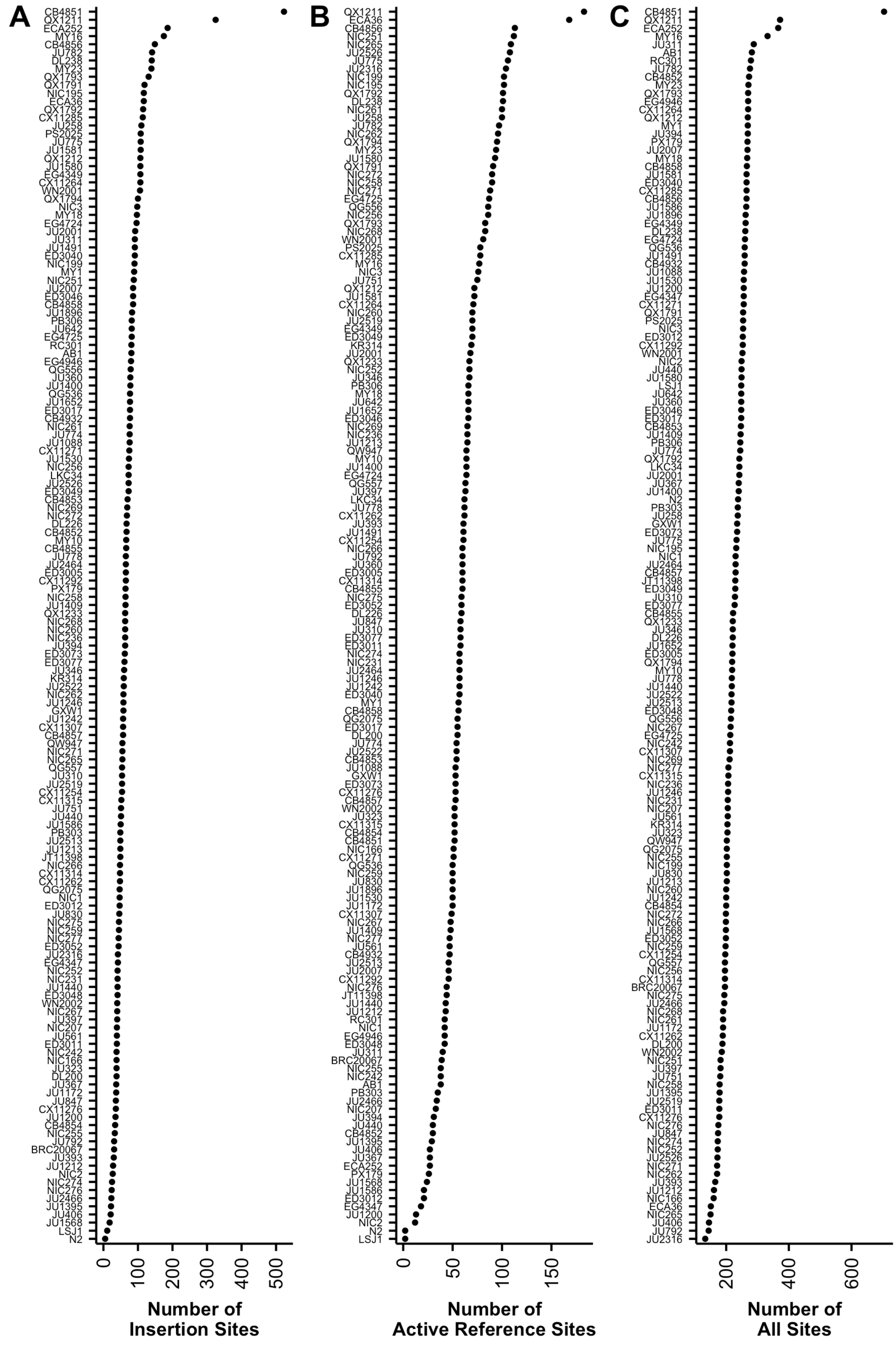

Supplemental Figure S4. Transposon sites per strain. The total number of A) insertion sites, B) active reference sites, and C) all transposon sites are shown per strain.

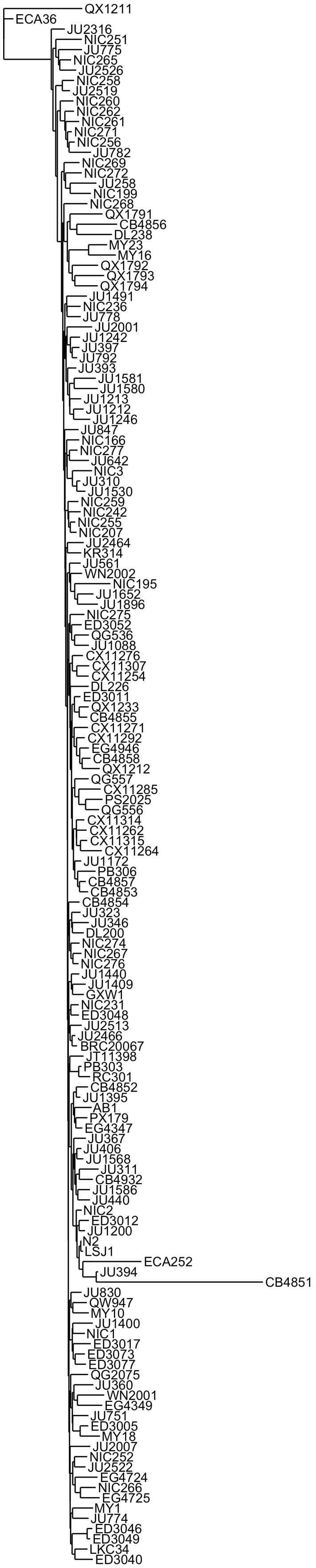

Supplemental Figure S5. Neighbor-joining tree based on transposon sites using QX1211 as the outgroup.

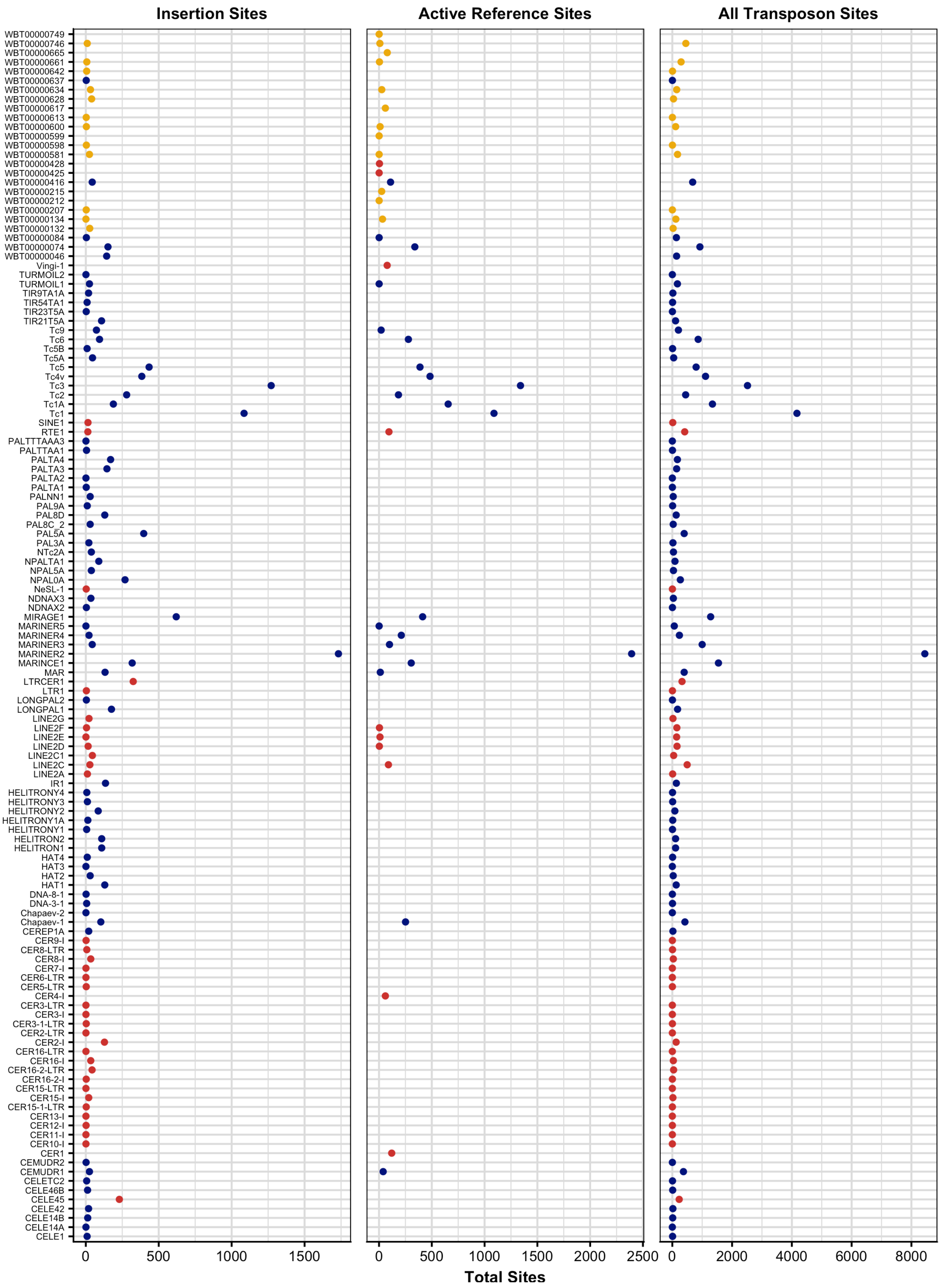

Supplemental Figure S6. Number of transposition sites per transposon family. Data represent total counts of transposition events for all 152 strains. Blue, red, and yellow points denote DNA transposons, retrotransposons, and transposons of unknown classification, respectively.

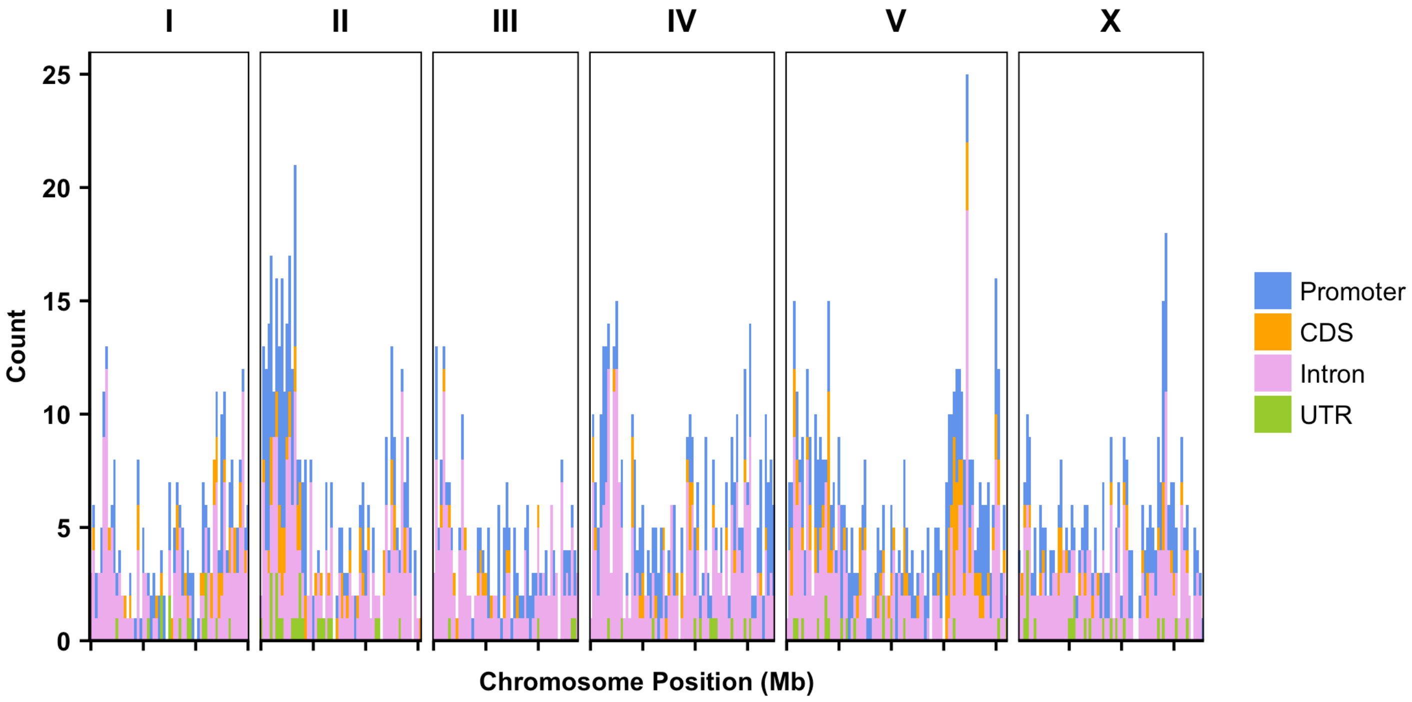

Supplemental Figure S7. Genomic locations of transposon insertions. The transposon insertion sites for TEs located in unique protein-coding genes are shown. The x-axis ticks mark every 5 Mb.

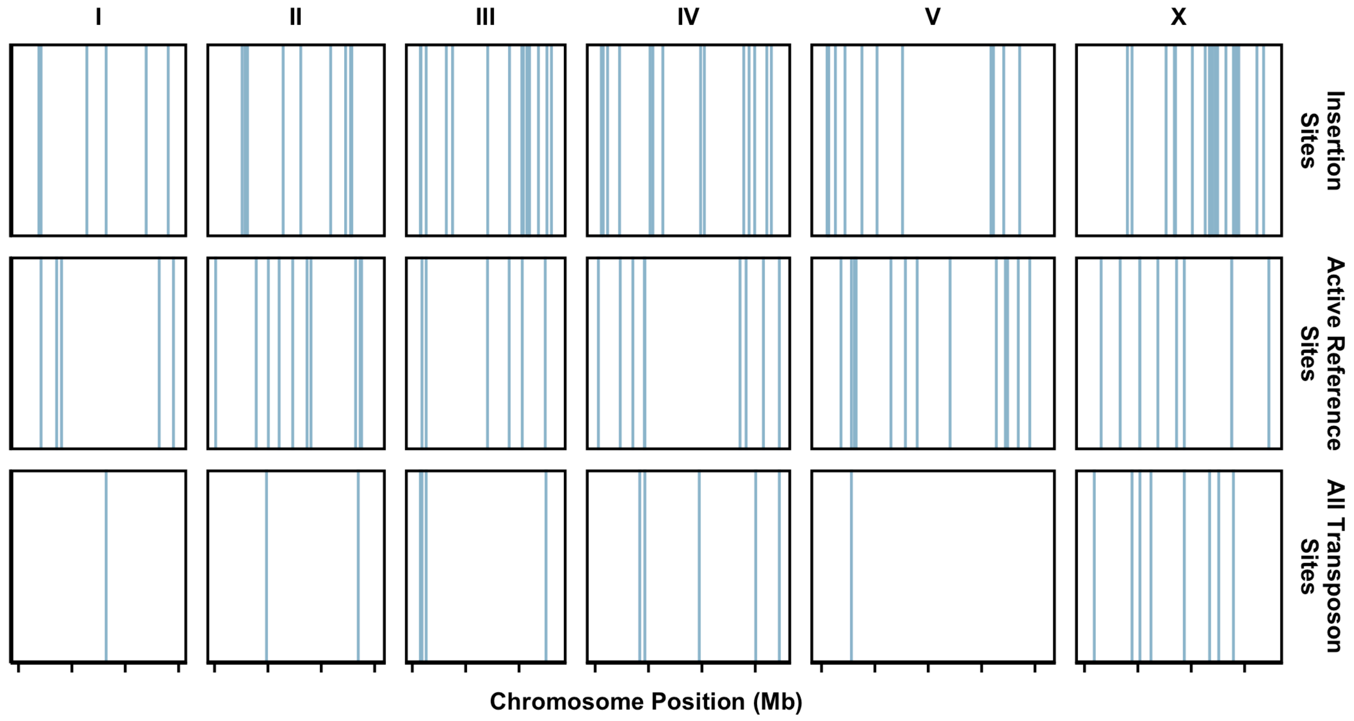

Supplemental Figure S8. Aggregate plot of each significant QTL faceted by TE insertions, active references sites, and all transposon traits. The location of each QTL from association mappings of transposon count traits is depicted by a vertical line. The x-axis ticks mark every 5 Mb.

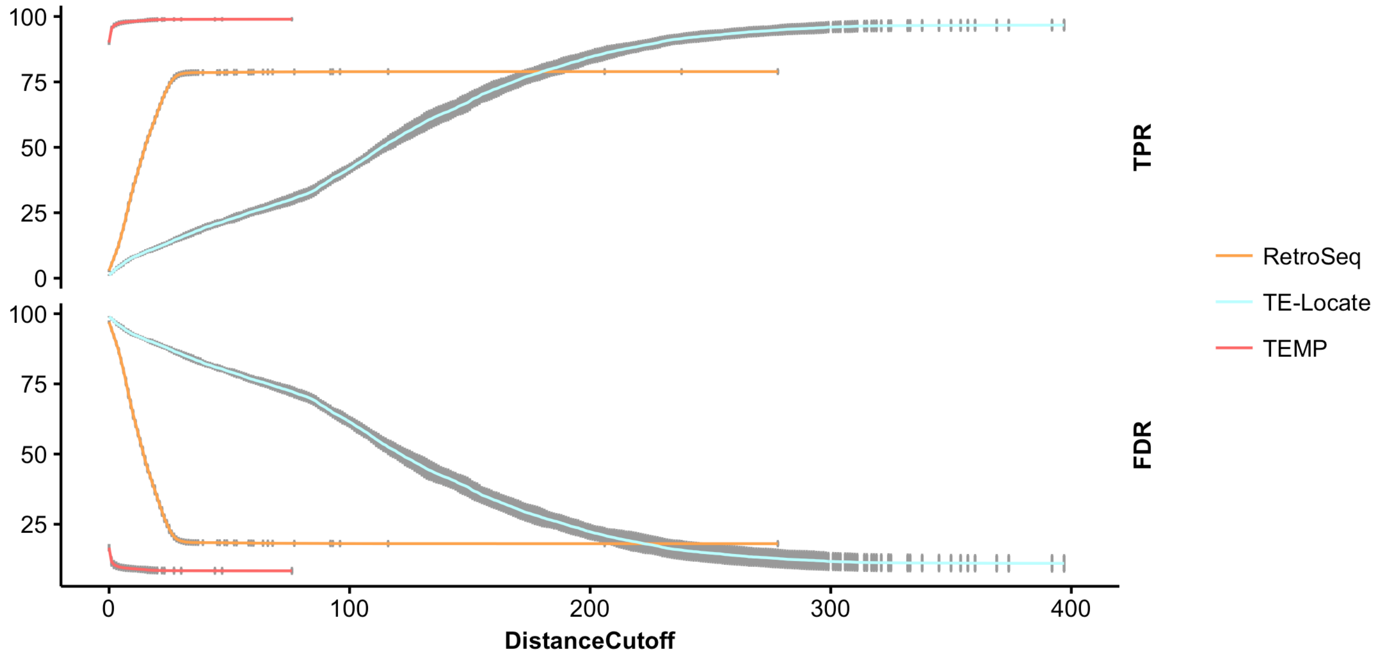

Supplemental Figure S9. Detection of transposon insertions for three programs. The average true positive rate (TPR) and average false discovery rate (FDR) shown are based on eight simulations. For each simulation, 1,000 transposons were inserted into the N2 BAM file. The performance of RetroSeq is shown in orange, TE-Locate in blue, and TEMP in red.

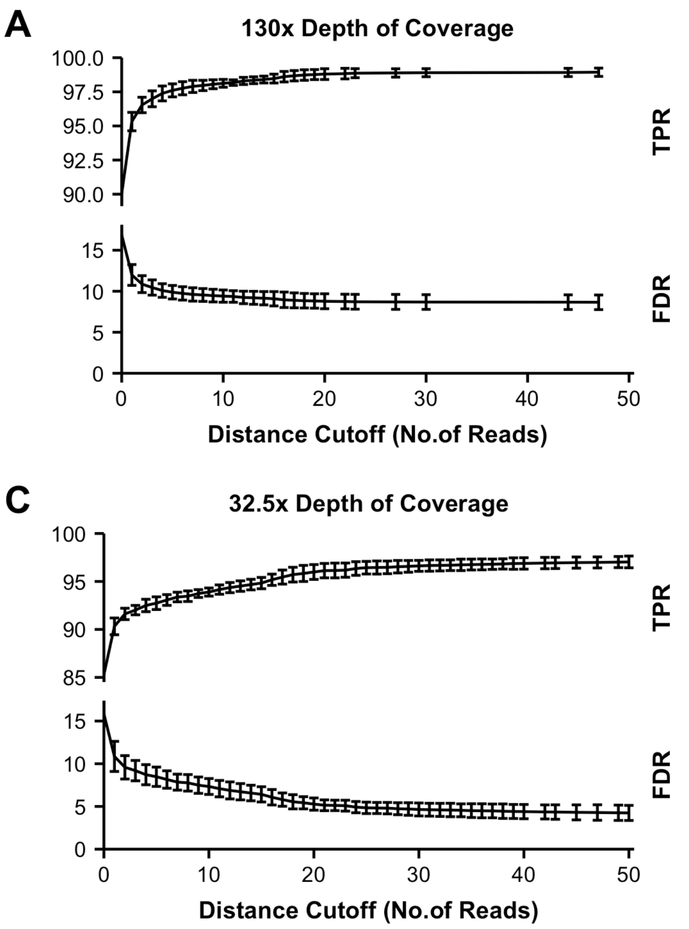

Supplemental Figure S10. Accuracy of the TEMP insertion caller across simulated coverage levels. The average true positive rate (TPR) and average false discovery rate (FDR) shown are based on eight simulations. For each simulation, 1,000 transposons were inserted into the N2 BAM file. The TPR was calculated at distance cutoffs ranging from 0 to 50 base pairs. Only detected transposons within the number of base pairs, denoted by the distance cutoff, from a simulated transposon were considered true positives. The increase in the TPR and decrease in FDR stabilize at approximately 20 base pairs. (A) through (D) use the same dataset but are down sampled to look at the effect of coverage on the TE calling parameter.

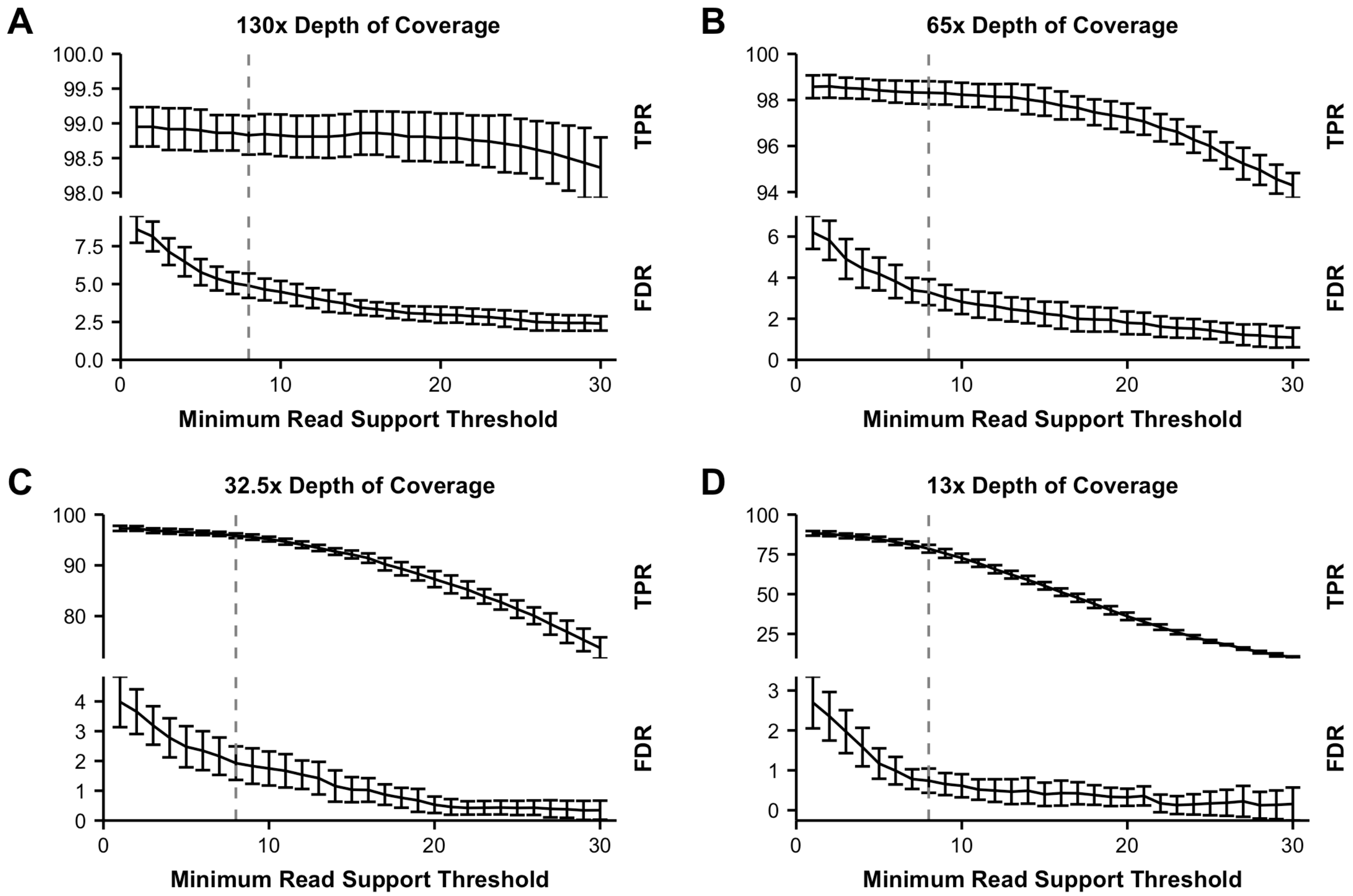

Supplemental Figure S11. Determination of minimum read support threshold for TEMP insertion caller across simulated coverage levels. The average true positive rate (TPR) and average false discovery rate (FDR) shown are based on eight simulations. For each simulation, 1,000 transposons were inserted into the N2 BAM file. Calls without the number of supporting reads denoted by the threshold level were discarded. The TPR was calculated at minimum read support thresholds ranging from 1 to 30 without requiring a minimum population frequency. To decrease the FDR without losing a large number of true positive calls, the minimum read support threshold was set to eight reads for the insertion caller, as denoted by the dotted vertical grey line. (A) through (D) use the same dataset but are down sampled to look at the effect of coverage on the TE calling parameter.

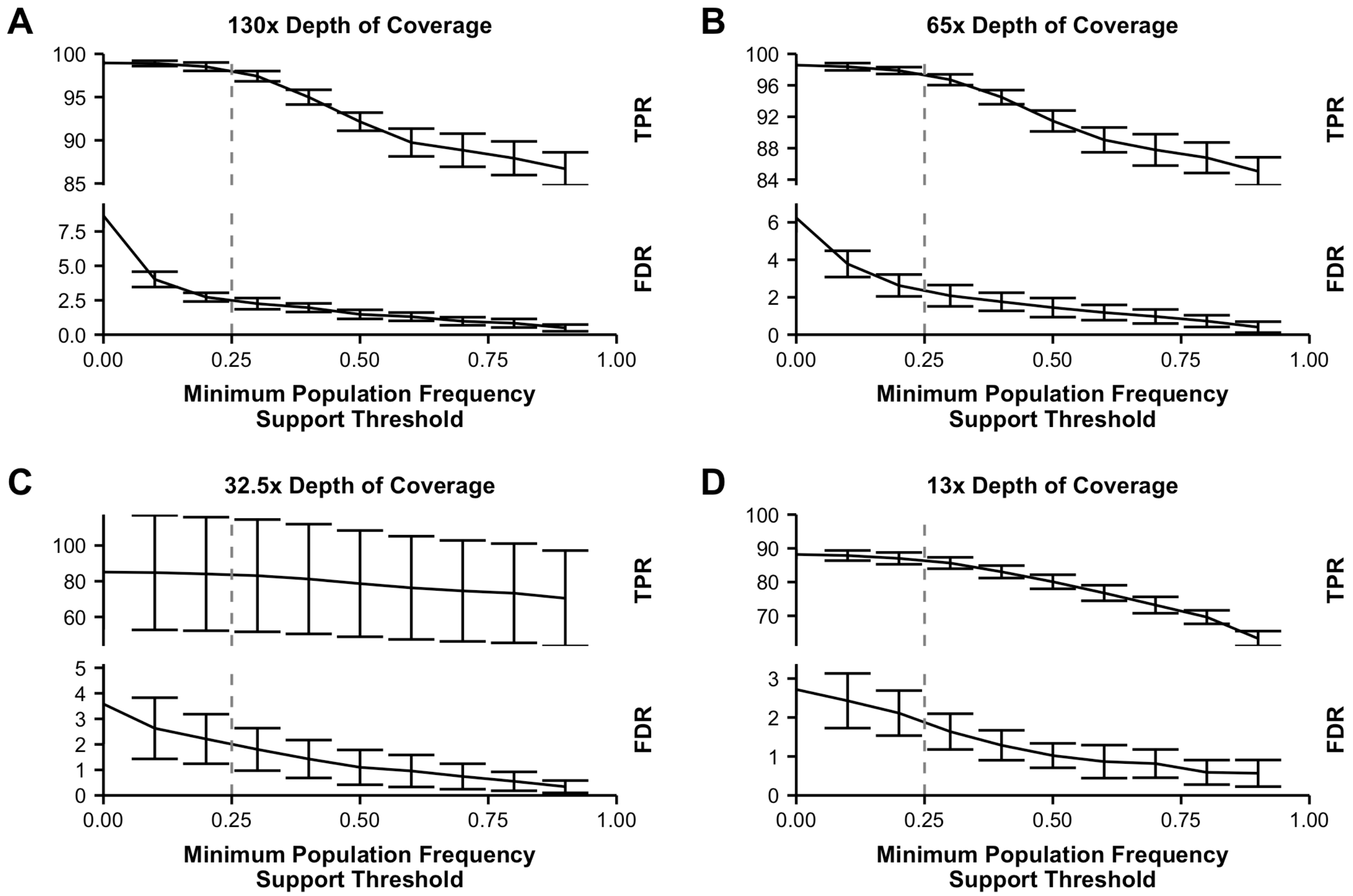

Supplemental Figure S12. Determination of population frequency threshold for TEMP insertion caller across simulated coverage levels. The average true positive rate (TPR) and average false discovery rate (FDR) shown are based on eight simulations. For each simulation, 1,000 transposons were inserted into the N2 BAM file. The population frequency represents the number of reads that support an insertion call out of the total number of reads at a given position. Calls below the threshold level were discarded. The TPR was calculated at population frequency thresholds ranging from 0.10 to 0.90 without requiring a minimum read support. To decrease the FDR without losing a large number of true positive calls, the population frequency threshold was set to 0.25 for the insertion caller, as denoted by the dotted vertical grey line. (A) through (D) use the same dataset but are down sampled to look at the effect of coverage on the TE calling parameter.

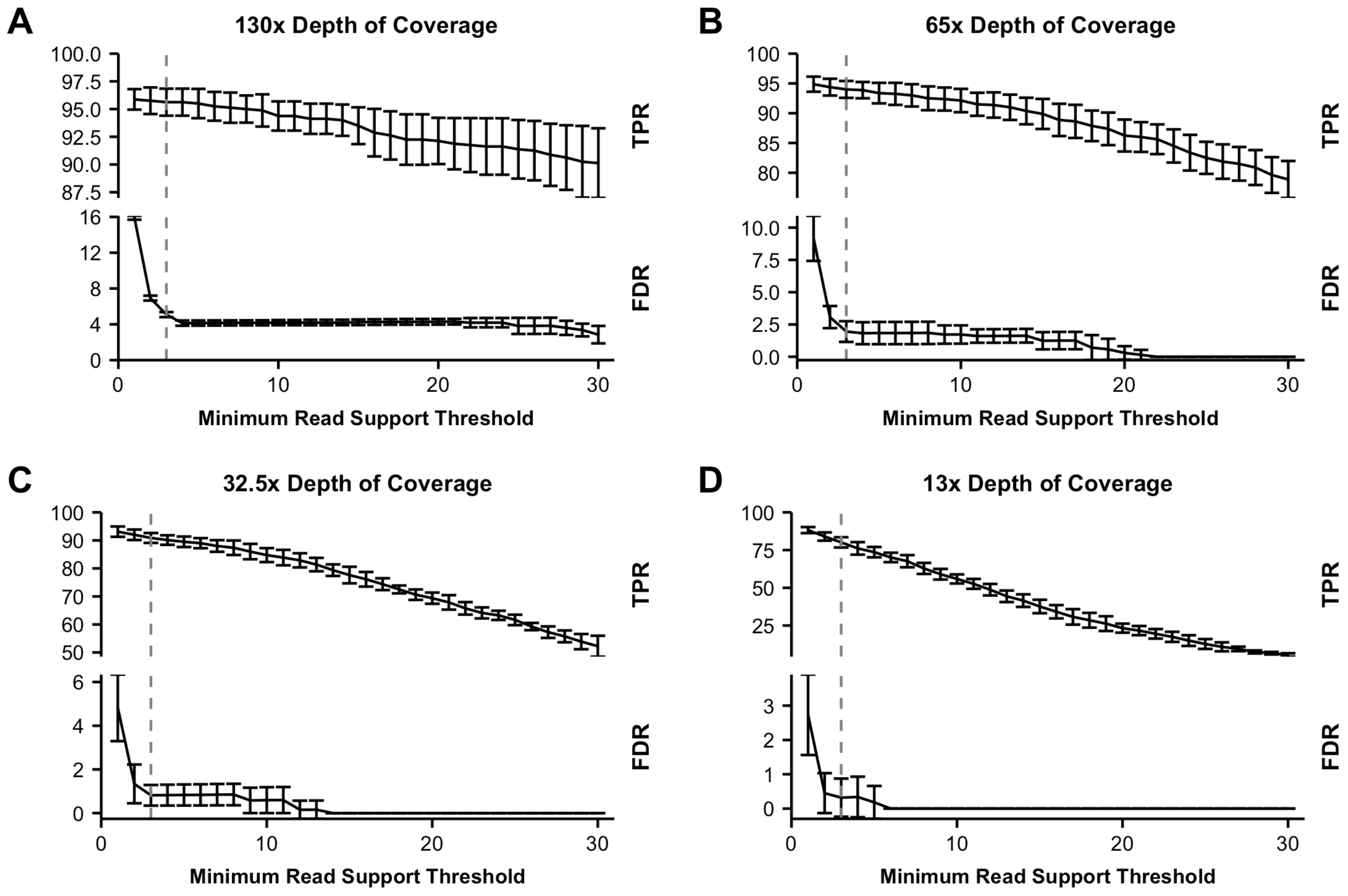

Supplemental Figure S13. Determination of minimum read support threshold for TEMP absence caller across simulated coverage levels. The average true positive rate (TPR) and average false discovery rate (FDR) shown are based on eight simulations. For each simulation, 100 transposons were inserted into the reference genome. Read support depicts the minimum number of reads needed that provided evidence for the absence call in order for that call to be considered a true positive. Calls without the number of supporting reads denoted by the threshold level were discarded. The TPR was calculated at minimum read support thresholds ranging from 1 to 30. To decrease the FDR without losing a large number of true positive calls, the minimum read support threshold was set to three reads for the insertion caller, as denoted by the dotted vertical grey line. (A) through (D) use the same dataset but are down sampled to look at the effect of coverage on the TE calling parameter.

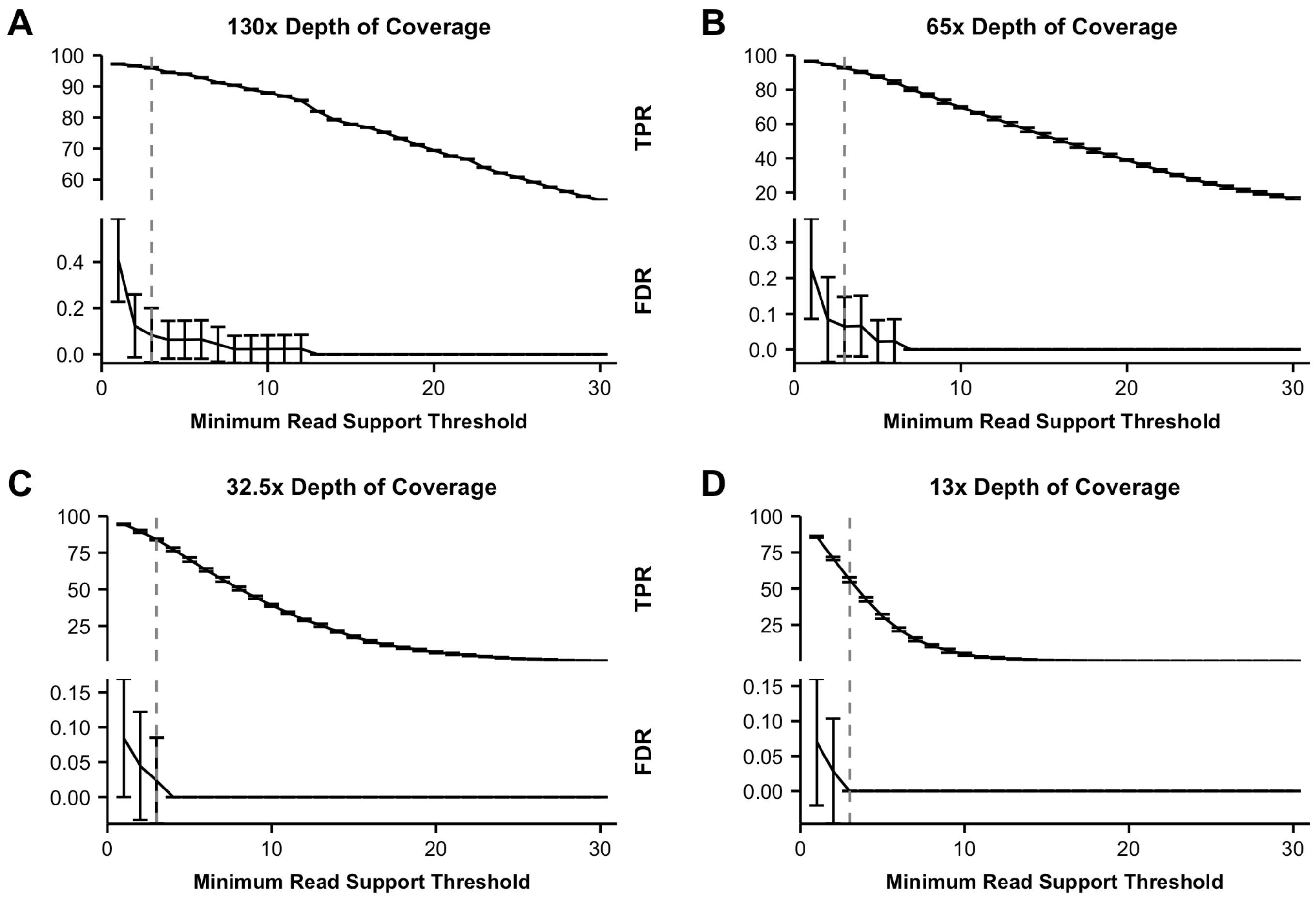

Supplemental Figure S14. Determination of minimum read support threshold for TE-Locate reference caller across simulated coverage levels. The average true positive rate (TPR) and average false discovery rate (FDR) shown are based on eight simulations. For each simulation, 100 transposons were inserted into the reference genome. Read support depicts the minimum number of reads needed that provided evidence for the absence call in order for that call to be considered a true positive. Calls without the number of supporting reads denoted by the threshold level were discarded. The TPR was calculated at minimum read support thresholds ranging from 1 to 30. To decrease the FDR without losing a large number of true positive calls, the minimum read support threshold was set to three reads for the insertion caller, as denoted by the dotted vertical grey line. (A) through (D) use the same dataset but are down sampled to look at the effect of coverage on the TE calling parameter.

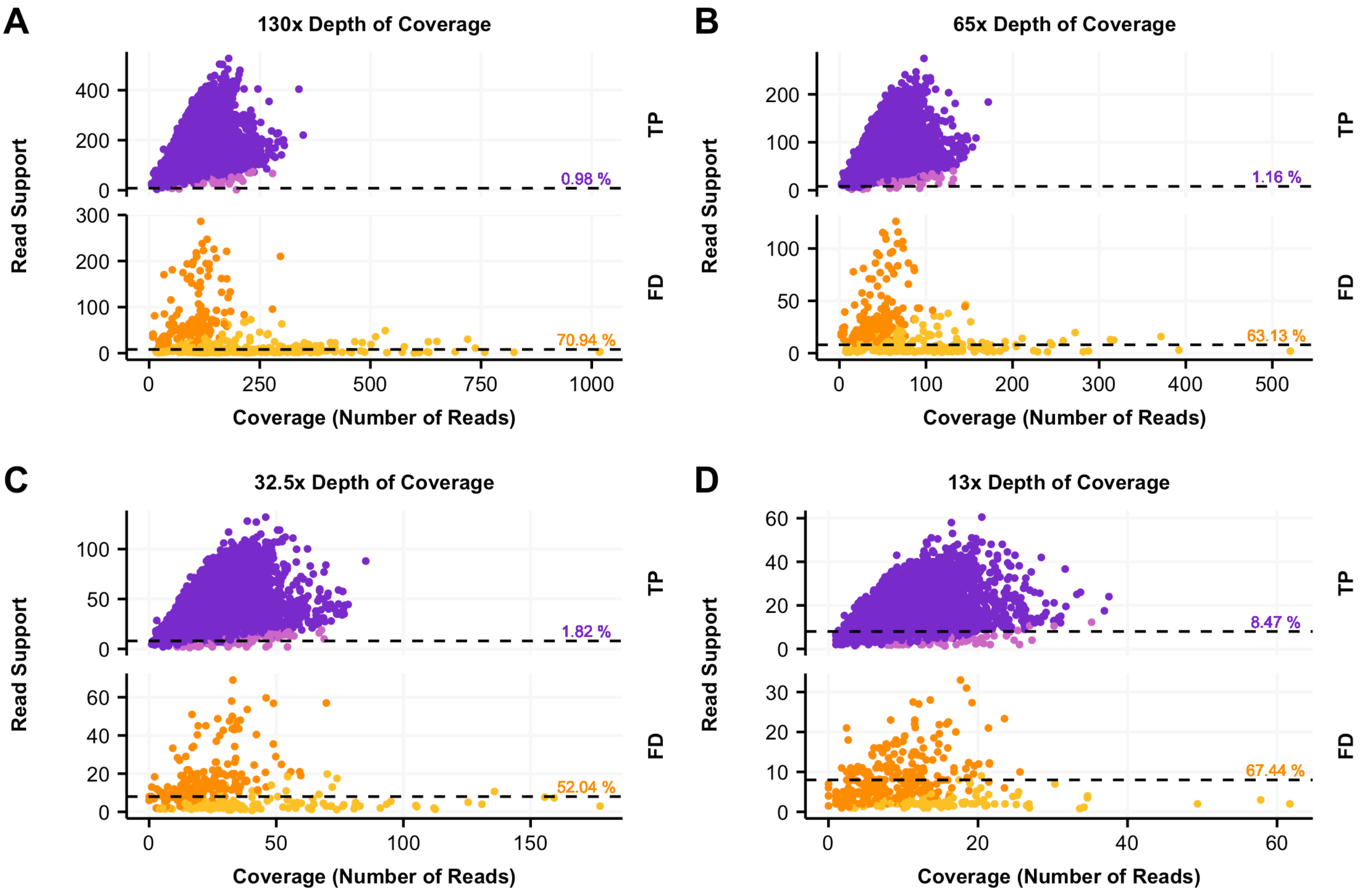

Supplemental Figure S15. Read support and coverage at sites of true positives and false discoveries for the insertion caller. Calls from all eight simulations are shown. True positive calls are shown in purple, and false discovery calls are shown in orange. Calls that are removed because they have a population frequency lower than 0.25 are shown in light purple and light orange, respectively. The dotted horizontal line marks the minimum read support threshold below which calls were discarded. The orange and purple percentages indicate the percentage of false discoveries and true positives discarded, respectively, after enforcing the minimum read support and population frequency thresholds. (A) through (D) use the same dataset but are down sampled to look at the effect of coverage on the TE calling parameter.

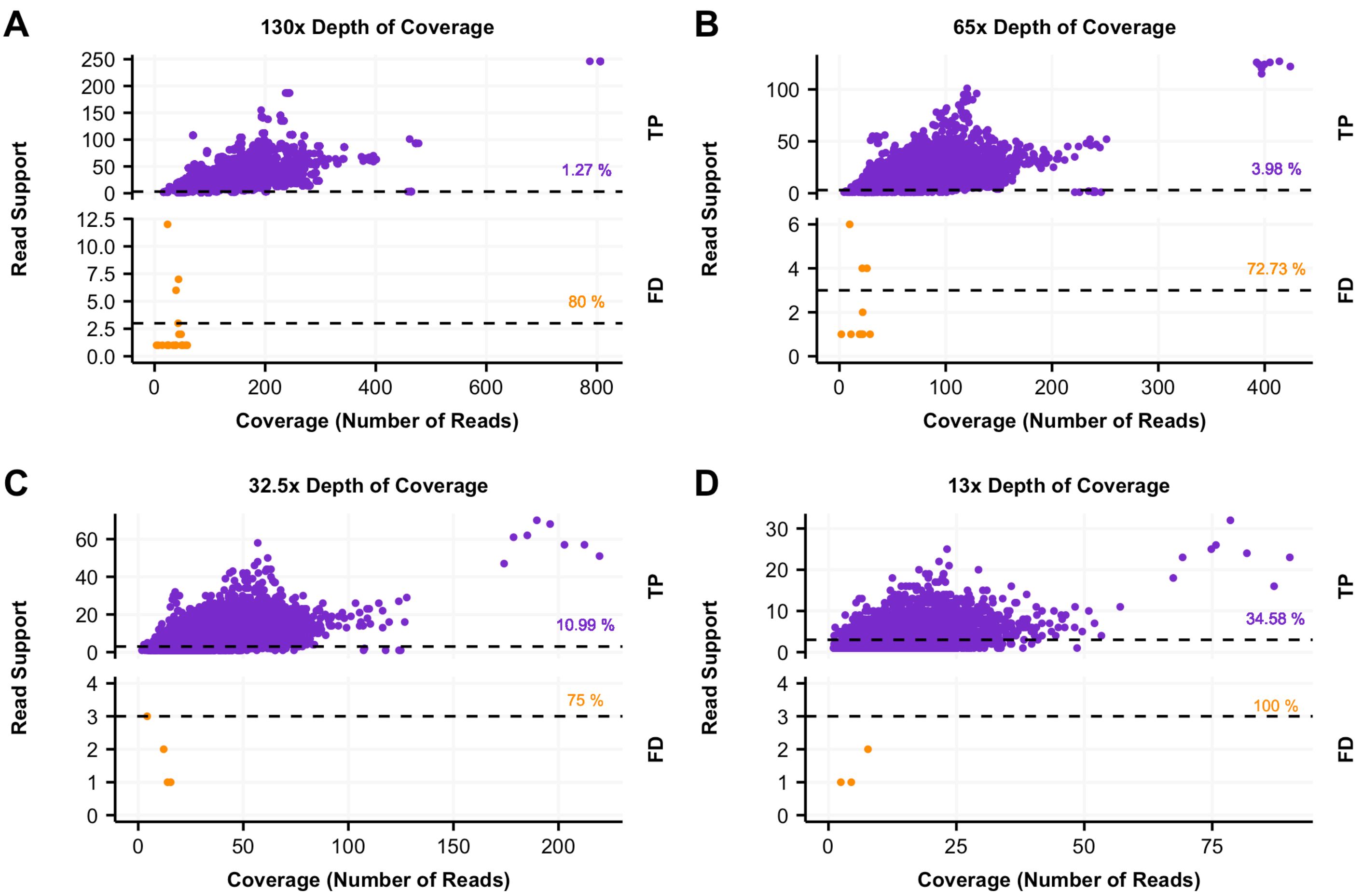

Supplemental Figure S16. Read support and coverage at sites of true positives and false discoveries for the reference caller. Calls from all eight simulations are shown. True positive calls are shown in purple, and false discovery calls are shown in orange. The dotted horizontal line marks the minimum read support threshold below which calls were discarded. The orange and purple percentages indicate the percentage of false discoveries and true positives discarded, respectively, after enforcing the minimum read support threshold. (A) through (D) use the same dataset but are down sampled to look at the effect of coverage on the TE calling parameter.

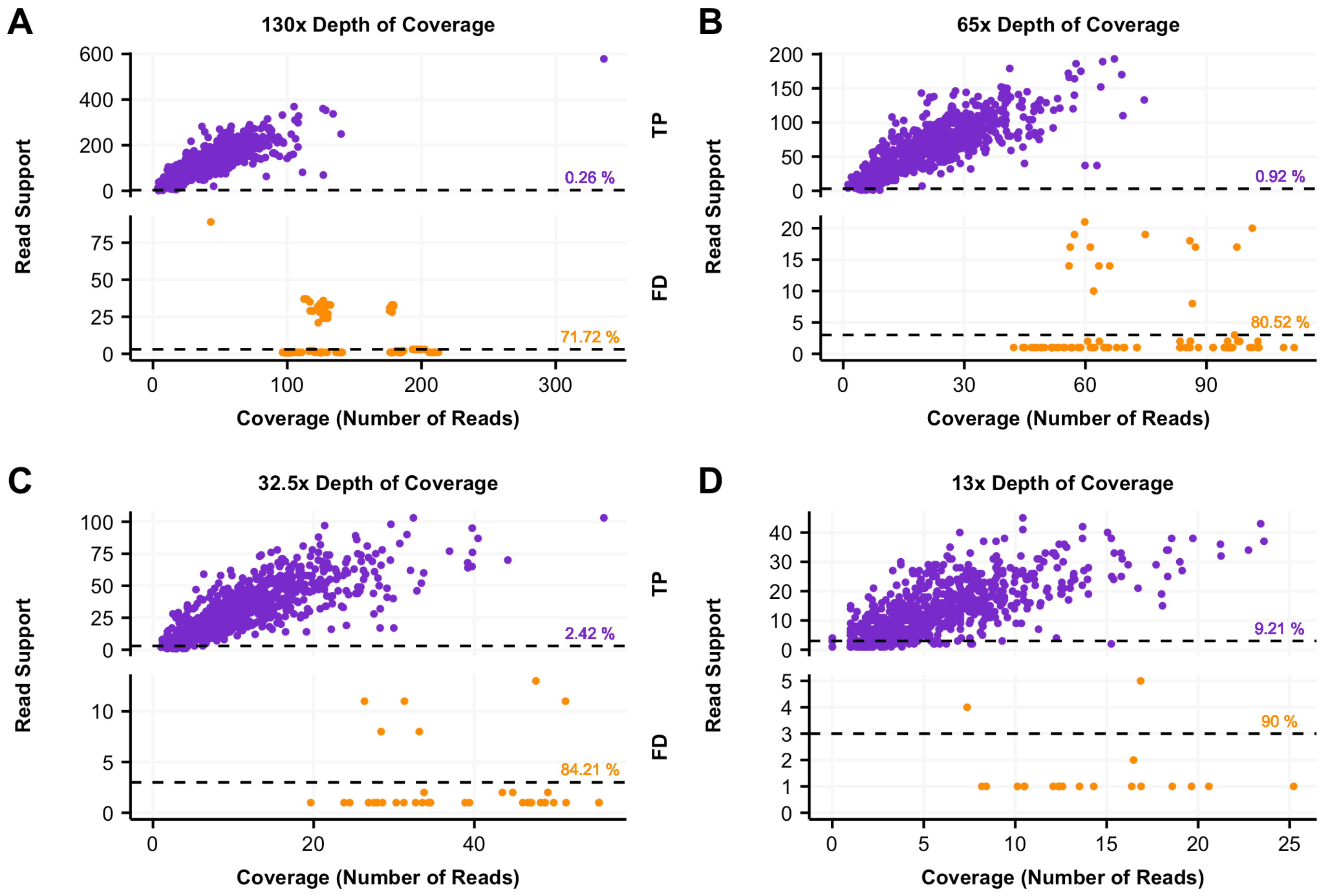

Supplemental Figure S17. Read support and coverage at sites of true positives and false discoveries for the absence caller. Calls from all eight simulations are shown. True positive calls are shown in purple, and false discovery calls are shown in orange. The dotted horizontal line marks the minimum read support threshold below which calls were discarded. The orange and purple percentages indicate the percentage of false discoveries and true positives discarded, respectively, after enforcing the minimum read support threshold. (A) through (D) use the same dataset but are down sampled to look at the effect of coverage on the TE calling parameter.

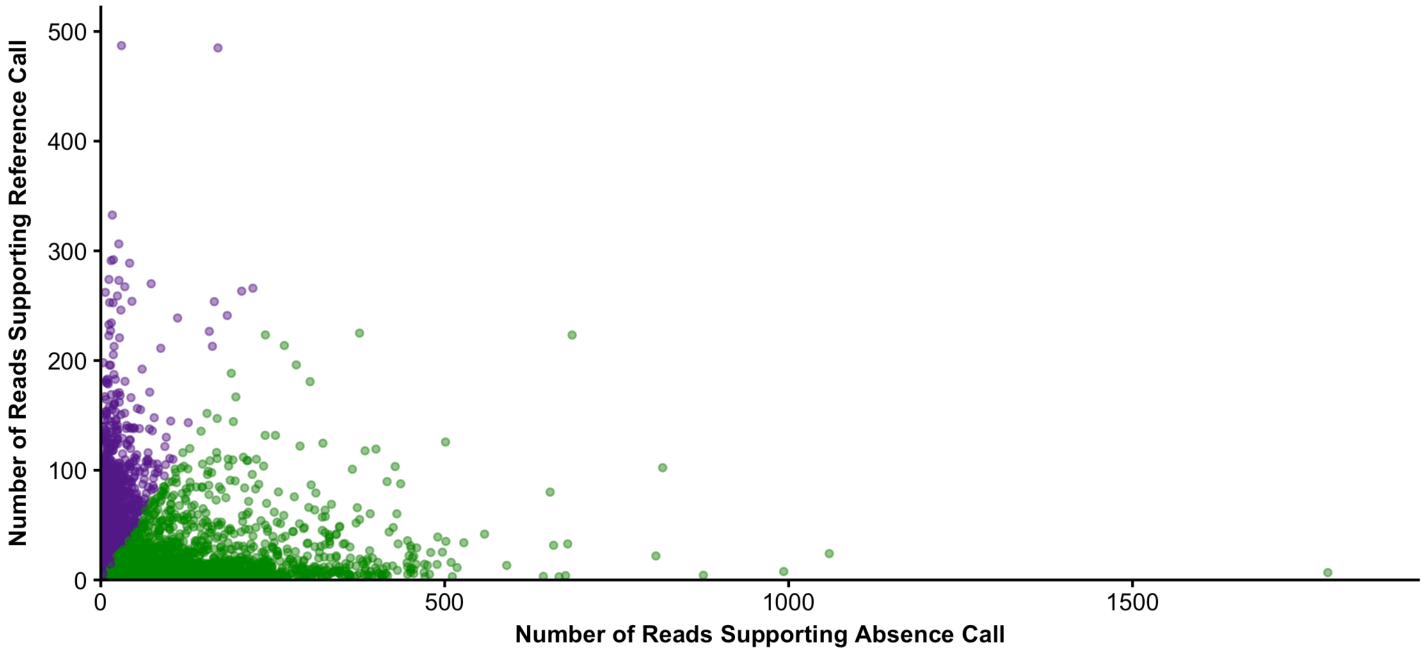

Supplemental Figure S18. Contradictory calls between the reference and absence caller. Each point represents a contradictory call in which reads supported both the presence and the absence of a particular transposon in any of the 152 strains. The transposon was classified as present or absent based on the caller with higher read support for the transposition event. The absence call was maintained when the number of supporting reads from the reference and absence call was equal. Purple represents calls with more reads supporting the presence of a transposon and green represents calls with more reads supporting the absence of a transposon.

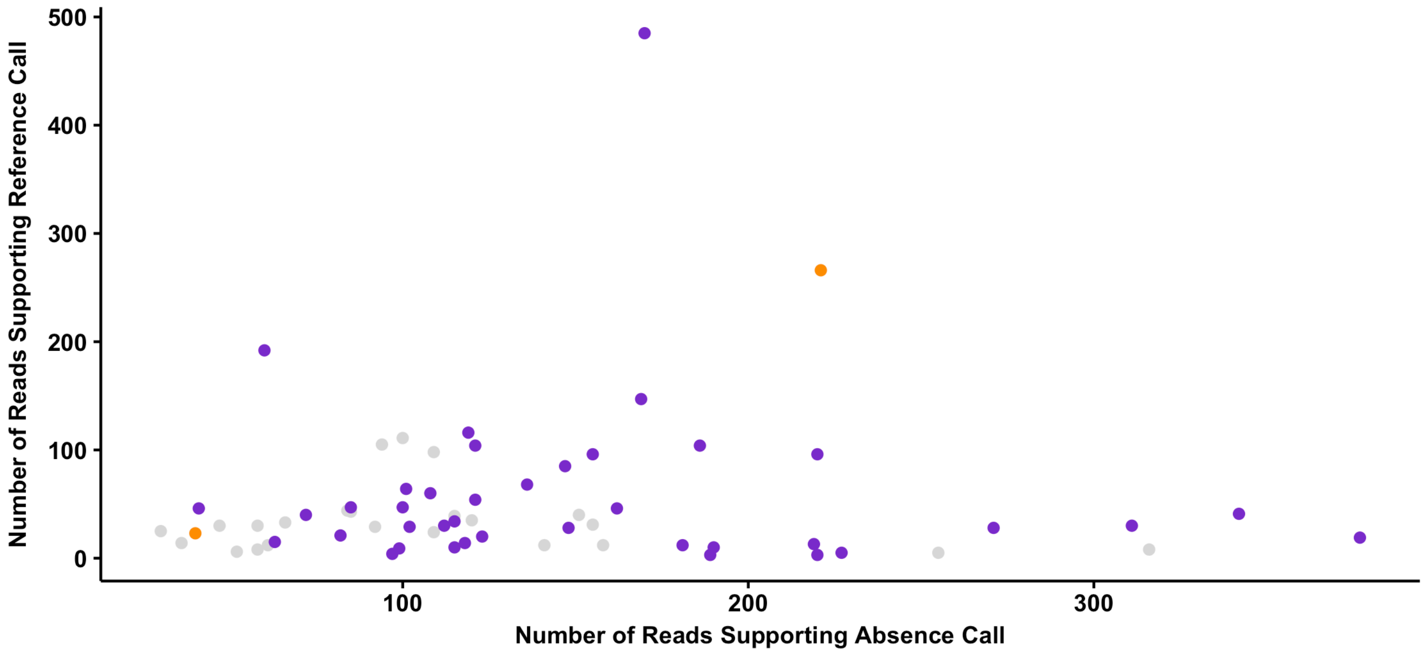

Supplemental Figure S19. Contradictory calls for the CER1 transposon. A total of 64 out of 152 strains were called both a reference and an absence for the CER1 retrotransposon at position III:8852596-8861460. Purple points are calls in agreement with the results of Andersen *et al.* 2012, orange points are calls that disagree with what was found in Andersen *et al.* 2012, and grey points are calls for a strain not present in Andersen *et al.* 2012. The final call was based on the caller with the highest read support, or on TEMP in instances of a tie in read support. Out of the 41 strains that could be compared to the results in Andersen *et al.* 2012, 39 were in agreement, suggesting that resolving contradictory calls based on read support is usually sufficient and no other filters are needed.

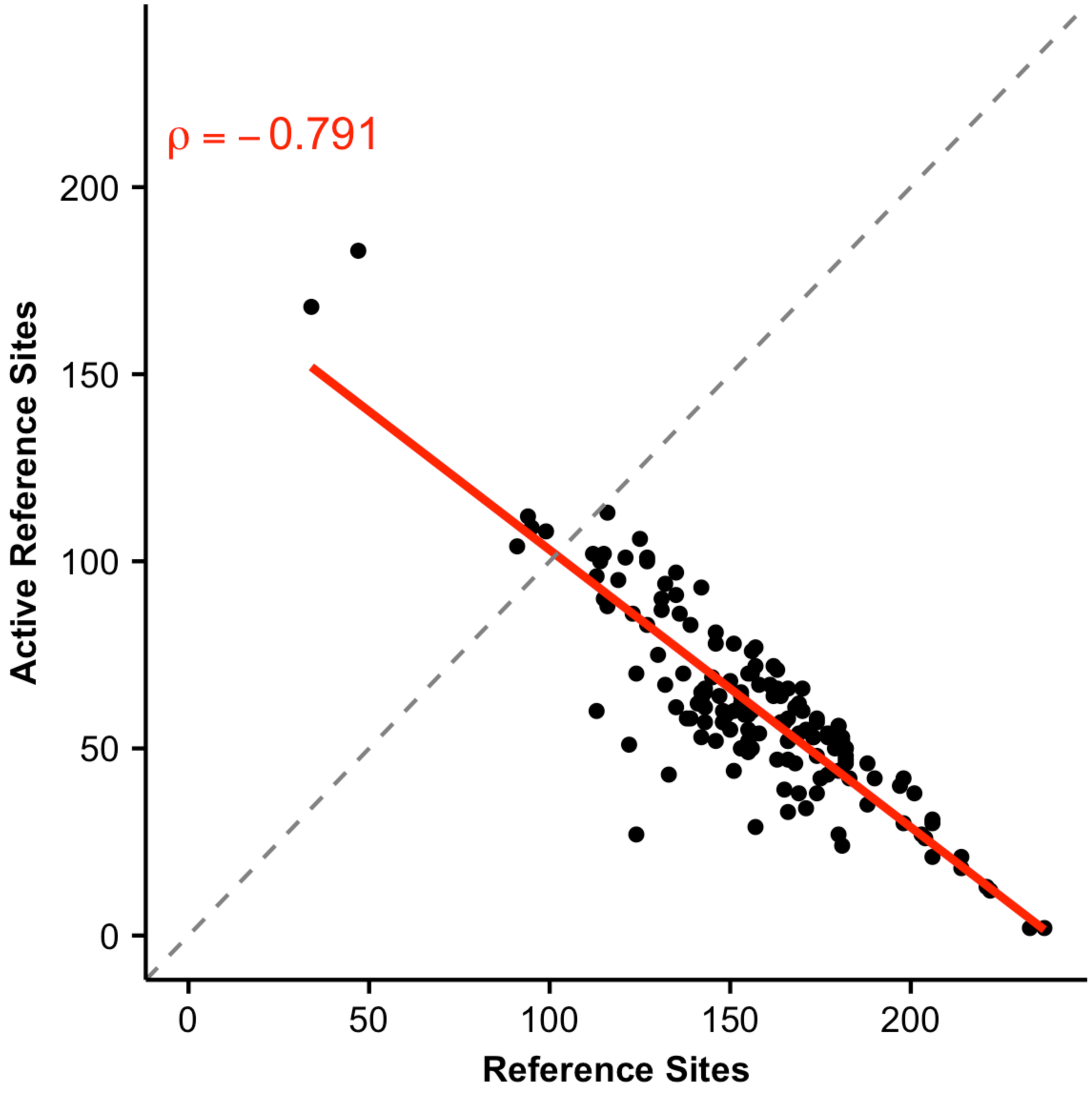

Supplemental Figure S20. Absence vs reference sites per strain. The number of transposon sites shared with the reference strain versus the number of active reference sites is plotted for each of the 152 strains. The identity line is shown in dotted grey, and the line of best fit is shown in solid red.

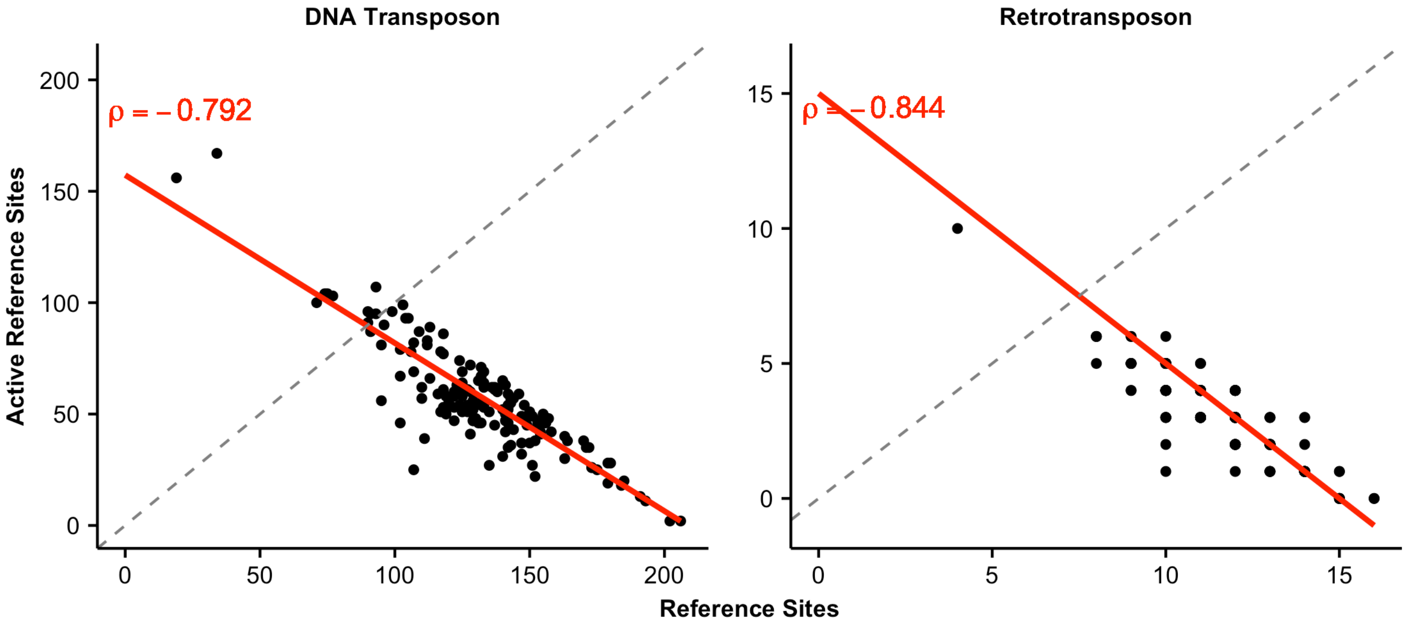

Supplemental Figure S21. Absence vs reference sites per strain. The number of transposon sites shared with the reference strain versus the number of active reference sites is plotted for each of the 152 strains. The identity line is shown in dotted grey, and the line of best fit is shown in solid red.
